# Accumbens connectivity during deep-brain stimulation differentiates loss of control from physiologic behavioral states

**DOI:** 10.1016/j.brs.2023.09.010

**Published:** 2023-09-19

**Authors:** Camarin E. Rolle, Grace Y. Ng, Young-Hoon Nho, Daniel A.N. Barbosa, Rajat S. Shivacharan, Joshua I. Gold, Dani S. Bassett, Casey H. Halpern, Vivek Buch

**Affiliations:** a Department of Neurosurgery, Perelman School of Medicine, University of Pennsylvania, Pennsylvania Hospital, Spruce Building 3rd Floor, 801 Spruce Street, Philadelphia, PA 19107, USA; b Department of Surgery, Corporal Michael J. Crescenz Veterans Affairs Medical Center, 3900 Woodland Ave, Philadelphia, PA, USA; c Department of Psychiatry and Behavioral Sciences, Stanford University School of Medicine, Stanford, CA 94304, USA; d Department of Neurosurgery, Massachusetts General Hospital and Harvard Medical School, 55 Fruit St, Boston, MA 02114, USA; e Department of Neurosurgery, Stanford University School of Medicine, 453 Quarry Road Office 245C, Stanford, CA 94304, USA; f Department of Neuroscience, University of Pennsylvania, 3700 Hamilton Walk, Richards D407, Philadelphia, PA 19104, USA; g Departments of Bioengineering, Physics and Astronomy, Electrical and Systems Engineering, Neurology, and Psychiatry, University of Pennsylvania, 210 S. 33rd St, Skirkanich Hall 240, Philadelphia, PA 19104, USA; h Santa Fe Institute, 1399 Hyde Park Rd, Santa Fe, NM 87501, USA

**Keywords:** Nucleus accumbens, Loss of control, Obesity, Binge-eating, Deep brain stimulation, Phase-locking value

## Abstract

**Background:**

Loss of control (LOC) eating, the subjective sense that one cannot control what or how much one eats, characterizes binge-eating behaviors pervasive in obesity and related eating disorders. Closed-loop deep-brain stimulation (DBS) for binge eating should predict LOC and trigger an appropriately timed intervention.

**Objective/hypothesis:**

This study aimed to identify a sensitive and specific biomarker to detect LOC onset for DBS. We hypothesized that changes in phase-locking value (PLV) predict the onset of LOC-associated cravings and distinguish them from potential confounding states.

**Methods:**

Using DBS data recorded from the nucleus accumbens (NAc) of two patients with binge eating disorder (BED) and severe obesity, we compared PLV between inter- and intra-hemispheric NAc subregions for three behavioral conditions: craving (associated with LOC eating), hunger (not associated with LOC), and sleep.

**Results:**

In both patients, PLV in the high gamma frequency band was significantly higher for craving compared to sleep and significantly higher for hunger compared to craving. Maximum likelihood classifiers achieved accuracies above 88% when differentiating between the three conditions.

**Conclusions:**

High-frequency inter- and intra-hemispheric PLV in the NAc is a promising biomarker for closed- loop DBS that differentiates LOC-associated cravings from physiologic states such as hunger and sleep. Future trials should assess PLV as a LOC biomarker across a larger cohort and a wider patient population transdiagnostically.

## 1. Introduction

Loss of control (LOC) eating, the subjective sense that one cannot control what or how much one eats [1,2], affects a large segment of the population worldwide, including nearly 25% of adolescents [3,4] and 10% of adults [5–8]. LOC eating is associated not only with disordered eating and obesity, but also with anxiety, depression, and worsened quality of life [9–16]. Unsurprisingly, LOC is a pervasive feature of binge eating that predisposes to obesity and is often refractory to treatment such as behavioral therapy and bariatric surgery [17–19]. Obesity then increases the risk of many comorbidities such as type 2 diabetes mellitus and cardiovascular disease, as well as premature death [20,21].

For patients with LOC that have treatment-refractory morbid obesity, deep brain stimulation (DBS) may be a promising next-line therapy that directly addresses food cravings. Ideally, a closed-loop or responsive DBS (rDBS) system would identify the onset of LOC (using a biomarker or another signal) and then provide an appropriately timed intervention to mitigate LOC. Prior work showed that LOC episodes were often preceded by cravings for preferred, rewarding food [22,23], particularly among individuals with binge eating disorder (BED) and individuals who were overweight or obese [24]. In search of biomarkers for LOC-associated cravings that could be used to guide rDBS, Wu et al. [25] found that low-frequency delta power peaks in the NAc were associated with food reward anticipation. Building on this work and on early studies showing that DBS of the NAc attenuated binge eating and induced weight loss [26–29], the BITES first-in-human early feasibility trial evaluated rDBS in the bilateral ventral NAc as a treatment for LOC eating (NCT03868670) [30]. Preliminary findings from the first two subjects are reported in Shivacharan et al. [31]; this study showed that, in both subjects, low-frequency power was increased in the bilateral ventral NAc before LOC eating compared to control periods. When the RNS device stimulated in response to brief increases in low-frequency power in the ventral NAc, there was a >80% reduction in LOC eating episodes. Although this low-frequency power signal detected LOC eating with high sensitivity (~70–80%), it had suboptimal specificity (~50–60%) [31].

As the BITES trial is ongoing, it is important to use early trial data to improve specificity and thus reduce inappropriate stimulation. We hypothesized that the poor specificity may be due to confounding between food craving and sleep; increases in low frequency NAc power have been shown during both food craving [25,31] and sleep [32–34].

We evaluate a measure of functional connectivity called phase-locking value (PLV) as a candidate biomarker that distinguishes LOC-associated craving from the physiologic states of sleep and hunger. PLV measures the level of information coordination between two network regions by computing the phase synchronization of two signals across trials [35,36]. We chose PLV as our connectivity metric for several reasons: 1) PLV is a well-validated metric shown to have behavioral relevance [37–41], 2) it is a computationally lightweight approach to measuring functional connectivity from intracranial EEG data [42], 3) unlike spectral coherence, PLV does not rely on the assumption of stationarity and thus is well-suited for neural data [43], and 4) PLV is robust to artifactual amplitude fluctuations [43].

For the first two subjects enrolled in the BITES clinical trial (NCT03868670) [30,31] we compare PLV across three types of behavioral conditions: craving (associated with LOC eating), hunger (associated with non-hedonic eating), and sleep. For one of the subjects, we also compare the craving condition with recordings obtained during normal, awake behavior. Our study is the first to evaluate inter- and intra-hemispheric NAc connectivity as a detector of LOC-associated cravings. We hypothesize that NAc PLV (a measure of functional connectivity) is able to differentiate LOC cravings from other physiologic states, such as sleep and hunger.

## 2. Materials and methods

### 2.1. Subjects

The BITES clinical trial is a multi-stage early feasibility study with staggered enrollment [30,31]. Two participants from the BITES clinical trial were enrolled in this study. All participant information, magnetic resonance (MR) and computed tomography (CT) imaging, local field potential (LFP), and behavior data were collected under Stanford University Institutional Review Board (IRB)-approved protocol (IRB #46563, NCT03868670).

### 2.2. RNS targeting and implantation

**Fig. 1.**
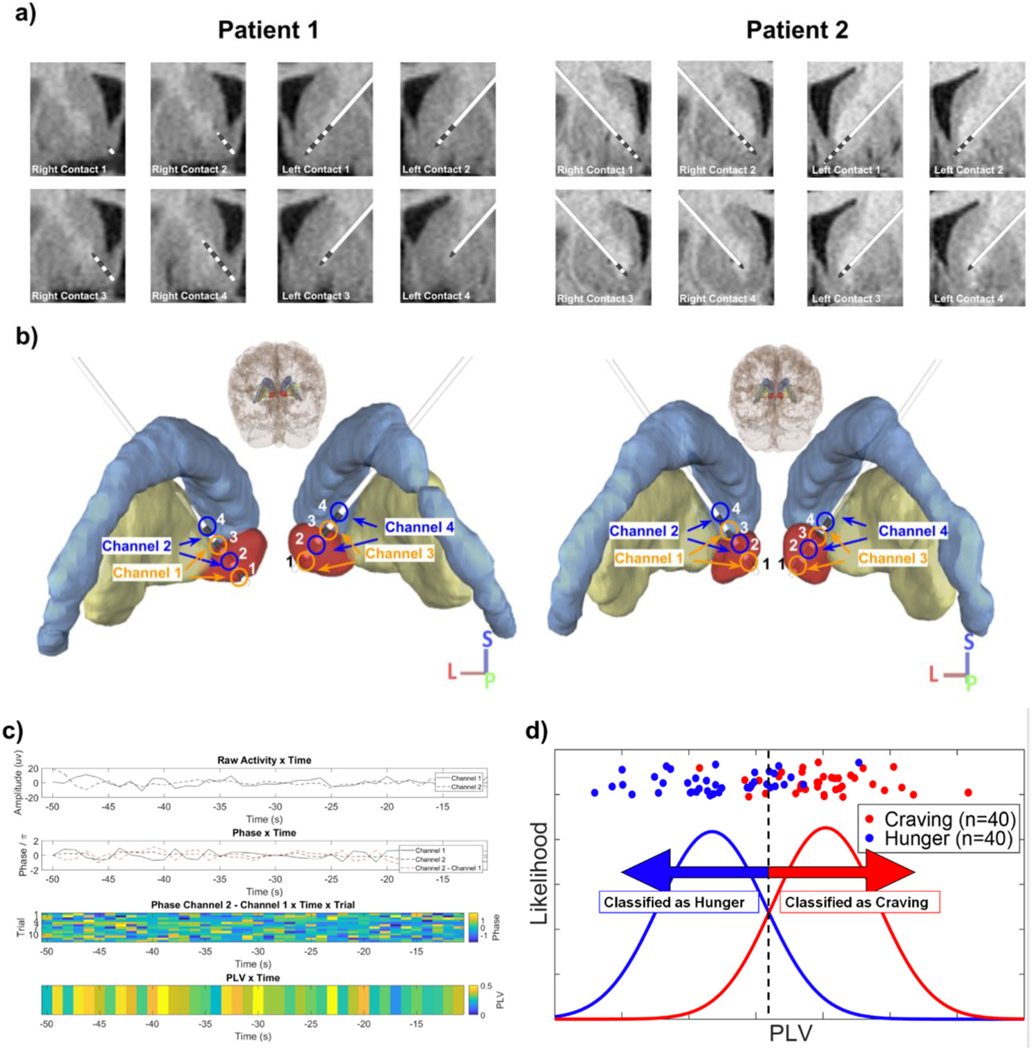
Experimental details. (a,b) The localization of the leads and contacts (quadripolar depth electrodes) placed bilaterally in the NAc for both subjects. Targeting of the electrode trajectory within the NAc was accomplished using stereotactic coordinates with Fast Gray Matter Acquisition T1 Inversion Recovery (FGATIR) and T1 images. A representation of electrode location co-registered to preoperative T1 is visualized in panel (a) and a 3D rendering of contact location is visualized in panel (b). The differences between pairs of contacts were used to generate four bipolar channels (panel b). Image legend: NAc-Red, Putamen-Yellow, Caudate-Blue. Adapted from Shivacharan et al. [31]. (c) Example of pipeline to extract PLV. The top panel plots the raw LFP trace from two channels across time bins. The second panel down plots the phase of those two channels, and the phase difference of those two channels across time. The third panel down shows the phase difference for those two channels across time for multiple trials. The last panel plots the PLV estimate (which reflects synchrony of the phase difference across trials) across time for those two channels. d) Schematic of the maximum likelihood classifier that was used to classify PLV estimates as pertaining to the craving versus hunger conditions. A similar classifier was used to classify craving versus sleep conditions. (For interpretation of the references to colour in this figure legend, the reader is referred to the Web version of this article.)

Following screening, cranial neurostimulators attached to two depth leads (one per hemisphere, each with 4 contacts spaced 3.5 mm apart; NeuroPace, Inc.) were surgically implanted in the subjects, targeting the NAc bilaterally (Fig. 1). Stereotactic coordinates standard to ventral striatum/ventral capsule indirect targeting were used (see Table S1). These coordinates were then determined for the NAc regions of interest by direct targeting using Fast Gray Matter Acquisition T1 Inversion Recovery MRI. The pre-operative MRI anatomical images were co-registered to post-surgical CT scan using Advanced Normalization Tools (ANTs) [44] for electrode localization [45]. The electrode artifacts from the co-registered post-operative CT, the pre-operative MRI, and atlas-based regions of interest were loaded in DSI Studio for 3D rendering [46].

We reported recordings from four channels (left ventral, left dorsal, right ventral NAc, and right dorsal NAc); each channel was derived from the bipolar difference of two contacts (Fig. 1A). The neurostimulator (250 Hz sampling rate) was programmed with the high-pass filter set to 1 Hz and the low-pass filter set to 90 Hz.

### 2.3. Ambulatory assessment

Prior to implantation, subjects were trained by a psychiatrist to recognize cravings, LOC eating behaviors, and hunger-driven eating behaviors (when participants ate ‘normal’-sized meals without LOC). Both subjects found that episodes of craving were frequently followed by LOC eating. By contrast, episodes of hunger were followed by typical eating behavior (without LOC). Therefore, episodes of intense craving associated with LOC eating were termed “craving”, and episodes of hunger-driven eating were termed “hunger”. Subjects were instructed to swipe the magnet twice to record episodes of craving, and to swipe once to record episodes of hunger. LFP snapshots were also recorded at a scheduled time of day during sleep hours (“sleep”). One subject was also instructed to swipe the magnet at times of day when she was awake, relaxed, and without cravings (“awake”).

### 2.4. LFP data processing

#### 2.4.1. LFP pre-processing

All LFP data analyses were performed in MATLAB (R2017b, The Mathworks Inc., MA) using custom scripts built upon the FieldTrip Toolbox [47].

Electrophysiological data were recorded at 250 Hz. LFP data were processed as follows: 1) de-trending, 2) 60 Hz (and harmonics: 120 Hz, 180 Hz) notch filters, and 3) 1–90 Hz bandpass filter. Following pre-processing, 40 s of data (−50 to −10 s preceding the magnet swipe) were extracted for each trial. The 10 s prior to the recorded event were excluded for all trials (i.e., sleep, craving and hunger) to remove any potential artifact from movement during the magnet swipe. In a sensitivity analysis, we analyzed the full 60 s of data (−60 to 0 s preceding the magnet swipe) that were stored for each trial on the device.

#### 2.4.2. Computing spectral power

Spectral power estimates were computed for each frequency band using multi-taper frequency transformation with 1.9 spectral smoothing and discrete prolate spheroidal sequences (dpss) tapering across 50 ms time intervals. Power estimates were averaged across the 40s time interval.

#### 2.4.3. Computing phase-locking value

To compute PLV, we applied band-pass filters: delta 1–4 Hz, theta 4–8 Hz, alpha 8–12 Hz, beta 12–30 Hz, low gamma 30–60 Hz, and high gamma 60–90 Hz. We then applied the Hilbert transform to compute the instantaneous phase of a filtered signal. Using Equation (1), we considered data across all N trials to compute PLV at each time pointt:

$$PLV_{t}=\frac{1}{N}\left|\sum_{n=1}^{N}\exp\left(i\left[\varphi_{1}(t,n)-\varphi_{2}(t,n)\right]\right)\right|$$

where $\varphi_{1}\left(t,n\right)$ and $\varphi_{2}\left(t,n\right)$ are the phases of the transformed signals 1 and 2, and n is the trial [36].

We computed PLV across all trials for a given condition (craving, hunger, or sleep) and frequency band (delta, theta, alpha, beta, low gamma, and high gamma). Thus, for each condition and frequency band, a PLV matrix was constructed with dimensions of time-points (40) x channels (4) x channels (4), so that the (t,i,j) component of the PLV matrix was the PLV between a “connectivity pair” of channels i and j at time point t, computed across all trials for a given condition. Since the trials were 40-s long, there were a total of 40 PLV data points for each condition.

Raw PLV estimates were averaged into 1-s time bins prior to statistical analyses for the purpose of statistical comparison and visualization.

#### 2.4.4. Statistical comparisons of experimental conditions

PLV estimates were compared between craving versus hunger and craving versus sleep using independent sample *t*-tests. In a supplemental analysis, PLV was compared between craving and awake conditions. All analyses were performed on time-averaged PLV (averaged into 1-s time bins) for each subject, each frequency band, and each connectivity pair. Significance was determined using a p-value threshold of 0.002, which was determined by Bonferroni correction for multiple comparisons across all connectivity pairs (4 pairs) and frequency bands (6 bands) [48].

As a supplemental analysis, independent sample *t*-tests were also performed to compare spectral power in the craving versus sleep conditions. Comparisons of spectral power in the craving versus hunger conditions are reported in Shivacharan et al. [31].

As an additional supplemental analysis, the correlations between power spectral data and PLV data (both binned into 1-s epochs) were compared for each combination of channel (power data) and connectivity pair (PLV data) across all conditions, patients, and for high versus low frequency data.

#### 2.4.5. Classification of experimental conditions

Using Bayesian Decision Theory [49], we built maximum likelihood classifiers that use PLV to classify craving versus hunger and craving versus sleep conditions. The Bayesian classifiers assign each event to the class with the largest posterior probability. The posterior probability P that a feature x is in class $\omega_{n}$ is defined as:

(2) $$P\left(\omega_{n}|x\right)=\frac{P\left(\omega_{n}\right)P\left(x|\omega_{n}\right)}{P(x)}=\frac{Prior\;of\omega_{n}\times \;Likelihood\;of\;\omega_{n}}{Evidence}.$$

As an example, the posterior probability for the craving class given a PLV value is given in Eq. (3). Since each class had the same number of PLV datapoints (equivalent to the number of timepoints within any given trial), the prior probabilities of all classes (*P*(*craving*),*P*(*hunger*)_*,*_ etc.) are equal. *P*(*PLV*) does not depend on the class. Thus, the ratio between posterior probabilities is determined by the ratio in the likelihoods *P*(*PLV*|*craving*) and *P*(*PLV*|*hunger*) (Fig. 1c).


(3) $$P(craving|PLV)=\frac{P(craving)P(PLV|craving)}{P(PLV)}.$$

We built classifiers for craving versus hunger and craving versus sleep. Accuracy was assessed using 10-fold cross-validation. Separate classifiers were trained for each subject; no classifier was trained on data from both subjects. However, as a supplemental analysis, we built classifiers that were trained on one subject’s data and tested on the other subject’s data. We also built classifiers that were trained on power spectrum data instead of PLV data.

## 3. Results

For subject 1, there were n = 19 craving trials, 12 hunger trials, and 29 sleep trials. For subject 2, there were n = 71 craving trials, 36 hunger trials, 143 sleep trials, and 26 awake trials. PLV was computed for left ventral-left dorsal, left ventral-right ventral, right ventral-right dorsal, and left dorsal-right dorsal NAc pairs. *P*-values and *t*-values were extracted and reported in Tables S2 and 3. For simplicity, we only present visualizations and statistical comparisons for low frequency (delta, 1–4 Hz) and high frequency (high gamma, 60–90 Hz) bands.

### 3.1. Statistical comparisons of experimental conditions

#### 3.1.1. Differentiating craving from hunger conditions

**Fig. 2.**
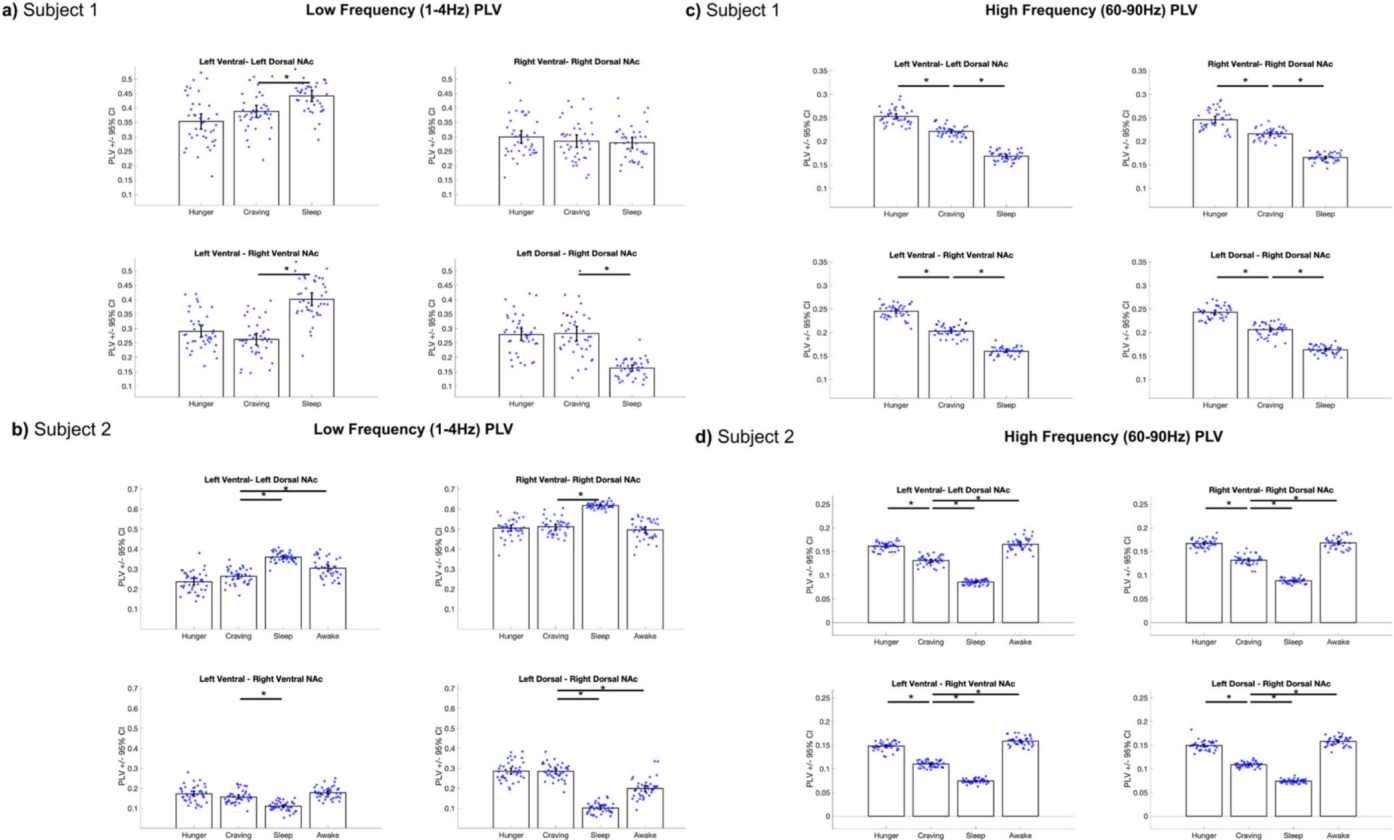
Phase locking value (PLV) estimates between conditions. PLV estimates for low frequency (1–4 Hz; Subject 1: panel a, Subject 2: panel b) and high frequency (60–90 Hz; Subject 1: panel c, Subject 2: panel d) bands across hunger, craving, and sleep conditions for left ventral-left dorsal, left ventral-right ventral, right ventral-right dorsal, and left dorsal-right dorsal NAc connectivity pairs. Red lines indicate significant differences below a p-value statistical threshold of 0.002 (Bonferroni corrected for 4 connectivity pairs x 6 frequency bands). Blue circles indicate trial-level data. (For interpretation of the references to colour in this figure legend, the reader is referred to the Web version of this article.)

For both subjects, PLV was significantly decreased for craving compared to hunger for all four connectivity pairs in the high gamma frequency band (Fig. 2c–d; *p <* 0.001 for all connectivity pairs), but no significant difference in the delta frequency band (Fig. 2a–b; *p >* 0.01 for all connectivity pairs). Total PLV per channel was significantly decreased in craving versus hunger conditions for all channels in the high gamma frequency band (*p <* 0.001 for all channels), but no significant difference in the delta frequency band (*p >* 0.01 for all channels) (Fig. S1).

#### 3.1.2. Differentiating craving from sleep conditions

PLV in the high frequency gamma band was significantly decreased for sleep compared to craving for all connectivity pairs in both subjects.

For both subjects, PLV was significantly increased for craving compared to sleep for all connectivity pairs in the high frequency gamma band (Fig. 2c–d; *p <* 0.001 for all connectivity pairs). In comparison, PLV in the low frequency delta band had an inconsistent direction of difference. For Subject 1, delta PLV was decreased for craving compared to sleep in left ventral-left dorsal and left ventral-right ventral, but increased in left dorsal-right dorsal (Fig. 2a; *p <* 0.001 for three connectivity pairs). For Subject 2, delta PLV was decreased for craving compared to sleep in left ventral-left dorsal and right ventral-right dorsal, but increased in left ventral-right ventral and left dorsal-right dorsal (Fig. 2b; *p <* 0.001 for all connectivity pairs).

Similarly, for both subjects, total PLV per channel was significantly increased in craving versus sleep for all channels in the high gamma frequency band (*p <* 0.001 for all channels), but only some channels in the low frequency delta band (Supplemental Fig. S1).

#### 3.1.3. Supplemental analyses

Subject 2 also had recordings during times when she was awake, relaxed, and without cravings (“awake”) to serve as a control condition. PLV was significantly decreased for craving compared to awake for all four connectivity pairs in the high gamma frequency band (Fig. 2d; *p <* 0.001 for all connectivity pairs), but only some of the connectivity pairs in the delta frequency band (Fig. 2b).

As a sensitivity analysis, we repeated the main analyses using the full 60 s of data available from each trial (instead of using only the middle 40 s of each trial) and found similar results (Fig. S2).

In additional supplemental analyses, we compared PLV (a measure of functional connectivity) with spectral power (a measure of local activity). Prior work demonstrated that low frequency power was higher for craving compared to hunger [31]. Here, we compared low frequency delta power in the craving versus sleep and craving versus awake states. We found that delta power was higher for sleep compared to cravings in bilateral ventral NAc (p *<* 0.001) but was not significantly different for bilateral dorsal NAc. There was no significant difference in delta power between cravings and awake in Subject 2 (Tables S4 and 5, Fig. S3).

We also investigated the temporal relationship between power spectral data and PLV data. There was no significant correlation between power and PLV for all behavioral conditions, subjects, and for high-frequency versus low-frequency data (*p* ≥ 0.0827, *rho* ≤ 0.242, *t* ≤ 1.47). Time-series of z-scored power and PLV data across time are plotted in Fig. S4 for visual comparison.

### 3.2. Classification of experimental conditions

**Fig. 3.**
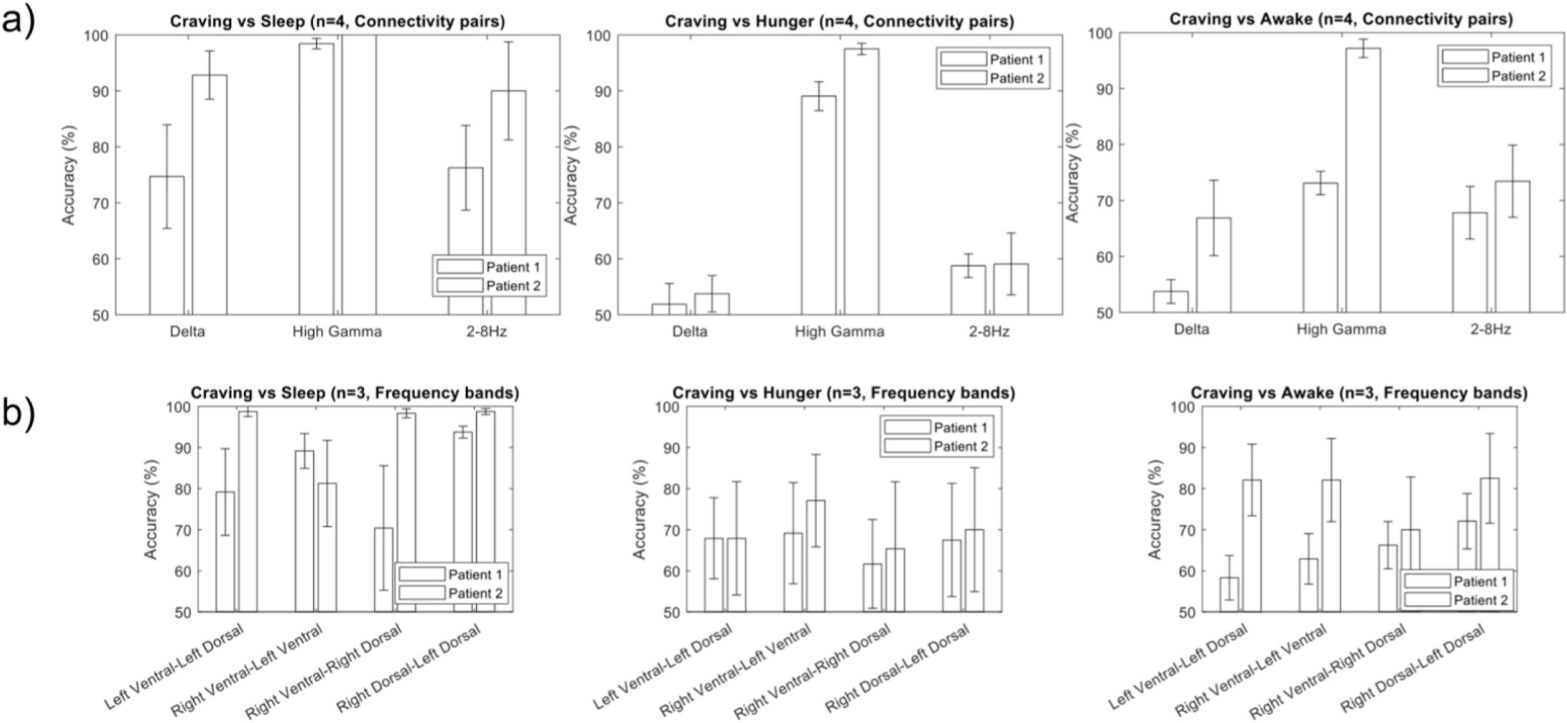
a) Maximum likelihood classifier results differentiating craving versus sleep, craving versus hunger, and craving versus awake; separate classifiers were trained for each frequency band and subject, and each classifier was trained on data from all four NAc connectivity pairs. b) Maximum likelihood classifier results differentiating craving versus sleep, craving versus hunger, and craving versus awake; separate classifiers were trained for each connectivity pair and subject, and each classifier was trained on data from all frequency bands.

We used maximum likelihood classifiers to predict craving versus hunger conditions and craving versus sleep conditions. For Subject 2, we additionally predicted craving versus awake conditions. Each of the conditions had n = 40 PLV datapoints (one datapoint for each second of the 40-s trial). In the first set of analyses, a classifier was trained for each subject and each frequency band, using PLV data from all four NAc connectivity pairs (Fig. 3a, Fig. S5, Table S6). In the high gamma band, classifiers achieved accuracies of 98.44 ± 0.94% and 100% for craving vs. sleep (Subjects 1 and 2, respectively), 89.06 ± 2.57% and 97.50 ± 1.02% for craving vs. hunger, and 97.19 ± 1.64% for craving vs. awake (Subject 2 only). The sensitivity was 87.74 ± 3.38% and 96.93 ± 1.17% for Subjects 1 and 2, respectively, and the specificity was 90.78 ± 1.75% and 98.13 ± 1.20%.

In the second set of analyses, a classifier was trained for each subject and each connectivity pair, using PLV data from all frequency bands (Fig. 3b, Fig. S5, Table S7). The highest accuracy for craving vs. sleep was in the right dorsal-left dorsal classifiers for Subjects 1 and 2 (93.75 ± 1.44% and 98.75 ± 0.72%, respectively), for craving vs. hunger in right ventral-left ventral (69.17 ± 12.30% and 77.08 ± 11.23%), and for craving vs. awake in right dorsal-left dorsal (82.50 ± 10.90% for Subject 2).

#### 3.2.1. Supplemental analyses

A set of maximum likelihood classifiers was trained using PLV data from one patient and then tested on PLV data from the other patient. Accuracy values were near 50% (Table S8); these accuracies were lower than the accuracies achieved by the subject-specific classifiers presented earlier.

Lastly, a set of maximum likelihood classifiers was trained using power spectrum data (Tables S9–10). In the delta band, classifiers using spectral data from all four NAc connectivity pairs had accuracies of 52.04 ± 6.26% and 65.23 ± 1.43% for craving vs. sleep (Subjects 1 and 2, respectively), 55.47 ± 8.21% and 48.15 ± 5.62% for craving vs. hunger, and 58.25 ± 7.78% for craving vs. awake (Subject 2 only). These accuracies were lower than the accuracies achieved by the PLV- based classifiers.

## 4. Discussion

Our study provides the first network-level characterization of the NAc as it relates to craving conditions associated with LOC, compared to other physiologic conditions such as hunger and sleep. The current study measured NAc functional connectivity using PLV [36,50] and demonstrated its utility in differentiating craving from hunger and sleep. We found that PLV in the high gamma frequency band distinguished craving from hunger and distinguished craving from sleep. Furthermore, high gamma PLV distinguished craving from other awake, non-hungry states.

Previous studies have shown that increases in low frequency NAc power predict LOC [25,31]. Wu et al [25] provide evidence of a possible mechanism, in which delta oscillations influence the timing of NAc action potentials involved in reward anticipation, including those related to eating. Moreover, spike-field coherence was also confirmed using a task that provokes robust incentive in a rare human case. However, these power-based measures of LOC had low specificity [31], likely because increases in low frequency NAc power are not specific to LOC, but are also found during sleep [32–34]. Our supplemental analyses delved into this hypothesis by directly comparing low frequency power in the RNS-implanted patients during LOC-associated cravings versus sleep. We showed that power is increased during sleep compared to cravings in the ventral NAc bilaterally. The directionality of this effect makes programming a craving-specific criteria for an RNS detector very difficult, as “elevated” low frequency power in the NAc could signal either craving or sleep.

With the aim of increasing the specificity of LOC detection, we examined PLV as a candidate biomarker for LOC. We analyzed ambulatory electrophysiology recordings acquired in two subjects with LOC and showed that PLV in the high gamma frequency band differentiated LOC cravings from the physiologic conditions of hunger and sleep. We additionally showed that high-gamma PLV differentiated craving from an awake, non-hungry control condition in Subject 2. Statistical comparisons (independent sample *t*-tests) demonstrated that PLV in the high frequency gamma band (60–90 Hz) is significantly different across conditions, with hunger and awake states resulting in the strongest gamma connectivity, LOC-associated cravings resulting in moderate gamma connectivity, and sleep resulting in the weakest gamma connectivity. Maximum likelihood classifiers used PLV values to differentiate LOC craving versus hunger and LOC craving versus sleep conditions with high accuracy, sensitivity, and specificity, particularly when using data from the high frequency gamma band (60–90 Hz). In both the *t*-test and classifier analyses, high-gamma PLV out-performed delta spectral power in differentiating between behavioral conditions. These findings suggest that PLV is more specific than spectral power in isolating the LOC behavioral condition.

The differences in PLV between the craving versus hunger and sleep conditions were consistent across all four connectivity pairs in both subjects. This finding suggests that high frequency PLV may serve as a biomarker for cravings that is robust to small variations in the anatomic placement of leads, and/or to variations in NAc anatomy between individuals. By contrast, the increase in power during craving was detected primarily in the ventral NAc channels [31]. The ventral NAc (associated with the “shell” subdivision of the NAc) and dorsal NAc (associated with the dorsal “core” subdivision) have been shown to have cooperative but distinct roles in reward processing [51–53]. Changes in ventral NAc delta power preceding LOC may reflect instantaneous activity in the NAc “shell” network, and prior mouse work has demonstrated that DBS modulation of the NAc shell reduced binge eating while core modulation did not [26]. By contrast, changes in functional connectivity (e.g., PLV) between inter- and intra-hemispheric dorsal and ventral NAc may reflect broader network coordination in the shell and core that reduces inhibition and promotes rewarding food stimuli. Moreover, since functional connectivity often predicts how focal stimulation propagates through brain networks [54–57], our PLV findings may have implications for understanding the mechanisms by which rDBS stimulation reduces cravings.

It is important to note that the differences in functional connectivity between the hunger, craving, and sleep conditions had spectral specificity; only high frequency gamma connectivity differentiated between the three conditions. NAc gamma oscillations (both high and low gamma) have been shown to be foundational to the mesolimbic network, and dysfunction of NAc gamma phase synchrony is associated with anxiety-related behaviors [58,59]. In humans, synchronous oscillations within subcortical and cortical networks, including cross-frequency coupling between gamma and lower frequency bands, have been linked to learning and decision making [60,61]. Further, high gamma frequency phase synchrony have been found to underlie the functional organization of NAc networks (particularly inter-NAc connectivity) during reward decision making [62–64]. This literature corroborates the significance of high frequency phase synchrony to NAc signaling, particularly as it relates to differentiating reward-based behavioral states (such as cravings in LOC).

We found that high gamma phase synchrony (as measured by PLV) effectively distinguished craving states from sleep, hunger, and other awake states. Although the PLV thresholds that distinguished these states were subject-specific (a classifier trained on one subject’s data was not able to consistently predict the other subject’s data), the relative differences in PLV were consistent across subjects. Both subjects had the lowest degree of phase synchrony during sleep, intermediate during craving, and the highest during hunger and other awake states. In concordance, the literature highlights the role of high NAc network connectivity in homeostatic hunger; [63,64], dopamine signaling in the NAc is involved in driving motivational, goal-directed responses to homeostatic needs, such as food-seeking behavior [63,65]. Specifically, NAc dopamine signaling in response to food cues is suppressed when subjects are satiated and exaggerated when subjects are hungry [65]. Therefore, it is not surprising that the current study found NAc connectivity to be heightened in states of hunger compared to states of craving (when hunger is absent). Additionally, this study found that sleep exhibited reduced NAc gamma connectivity compared to craving. Cognitive activity in the NAc during sleep does not appear to be indexed by high frequency connectivity, but rather by low frequency activity; prior work showed NAc delta power peaks during sleep [32], and our results showed elevated delta band connectivity during sleep.

The findings of this study are limited by the small sample size of two subjects and are further limited by small within-subject data samples. However, this first-in-human trial is ongoing, and thus, it is the authors’ intent to leverage all available data to study this promising novel intervention for food craving and beyond. Second, there is variation in the literature regarding the definition of low-frequency bands; some studies have focused on 2–8 Hz [31], while others [25] (including the current study) have focused on the delta band specifically (1–4 Hz). The differences in frequency band definition limit our ability to compare our findings to other studies about low-frequency NAc activity. Third, as is the case for all intracranial signal analyses derived from more than one patient, the lead anatomic locations varied slightly between the two subjects. Specifically, due to the anatomy of blood vessels, our first subject required a considerably more posterior entry point for the right lead compared to the left lead. Although the bilateral targets (and the most ventrally-located contact) are similarly located, the difference in entry points resulted in a considerably more posterior dorsal NAc contact on the right side compared to the left for this subject. In contrast, the anatomy of our second subject allowed for more symmetrical lead placement, with dorsal and ventral NAc contacts similarly positioned in the antero-posterior axis for both leads. Such variations (in lead placement and anatomy) will likely be present in follow-up studies with larger subject samples. Larger sample sizes will be important for assessing the generalizability of these findings to broader subject populations, and in better characterizing inter-individual differences and assessing the need for personalized management of cravings associated with LOC eating.

## 5. Conclusions

Our findings suggest that NAc connectivity is a promising candidate biomarker for craving associated with LOC eating. Future work should evaluate this biomarker in a larger and more diverse patient sample.

## Supplementary Material

1

## Abbreviations

NAc: Nucleus accumbens
PLV: Phase-Locking Value
LOC: Loss of Control
DBS: Deep Brain Stimulation
BED: Binge-Eating Disorder
