# Supplementary material for: Accumbens connectivity during deep-brain stimulation differentiates loss of control from physiologic behavioral states
Source: Brain Stimul. Author manuscript; Available in PMC 2024 Jan 26. (PMC10811591; doi:10.1016/j.brs.2023.09.010)
Supplement: 1 [file NIHMS1941405-supplement-1.docx]

**Supplemental Figures**


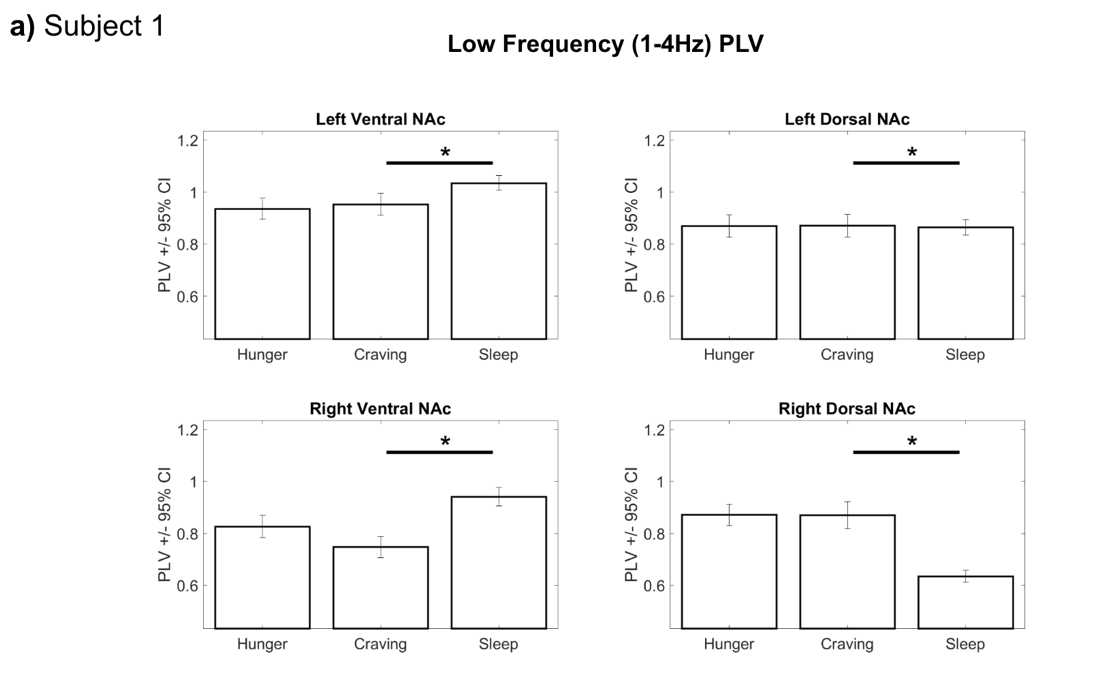

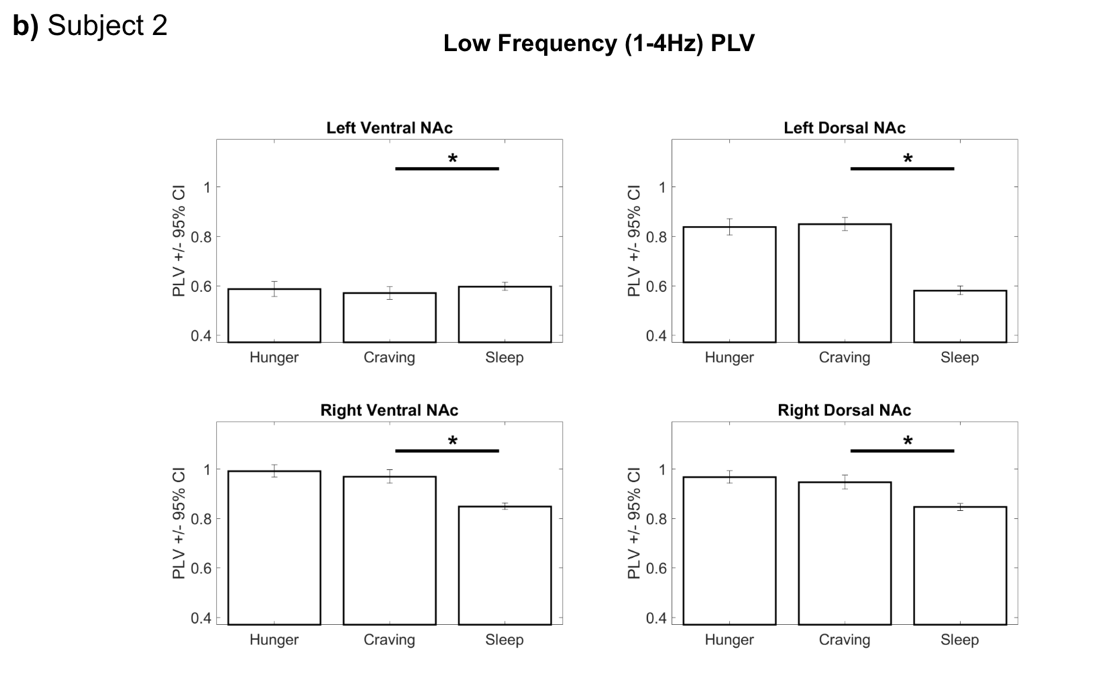

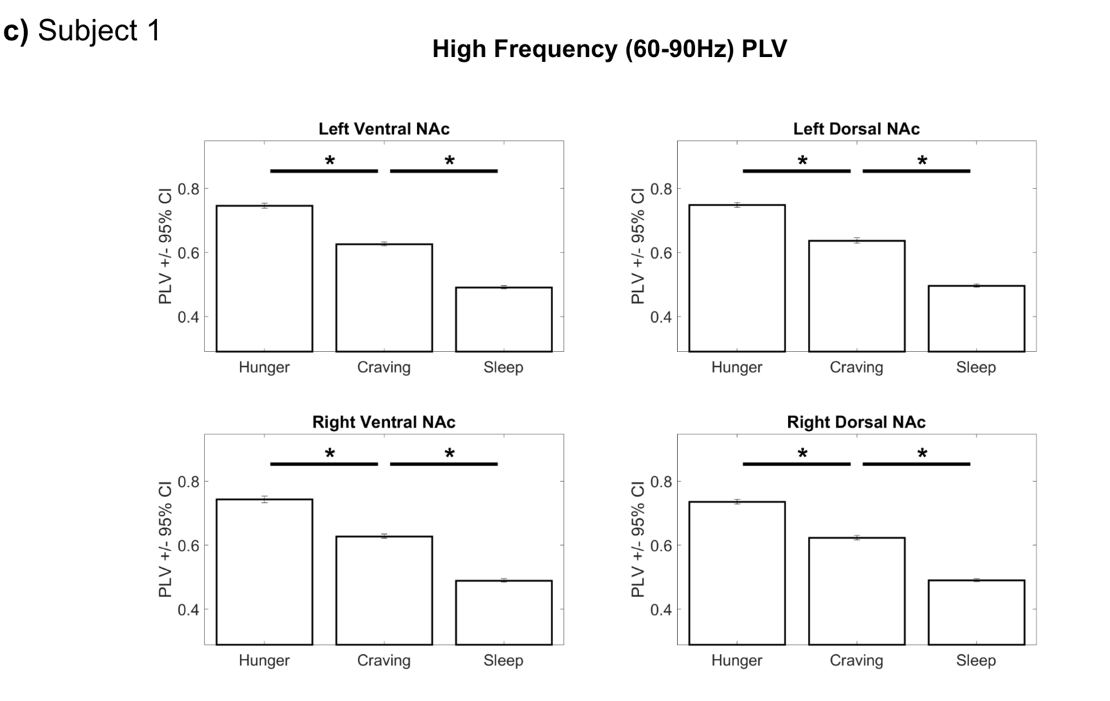

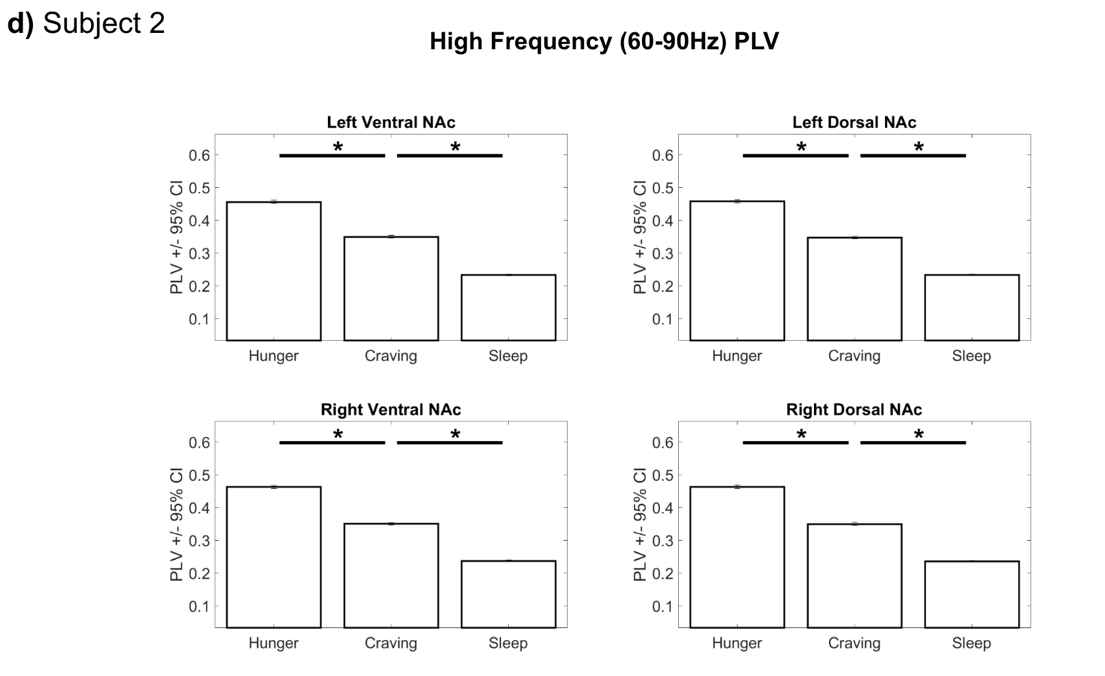


**Figure S1. Summed Phase Locking Value (PLV) estimates between conditions.** PLV estimates for low frequency (1-4Hz; Subject 1: panel a, Subject 2: panel b) and high frequency (60-90Hz; Subject 1: panel c, Subject 2: panel d) across hunger, craving, and sleep conditions for summed PLV at left ventral, left dorsal, right ventral, and right dorsal NAc channels. Red lines indicate significant differences below a statistical threshold of .002 (Bonferroni corrected for 4 “connectivity pairs” * 6 frequency bands).


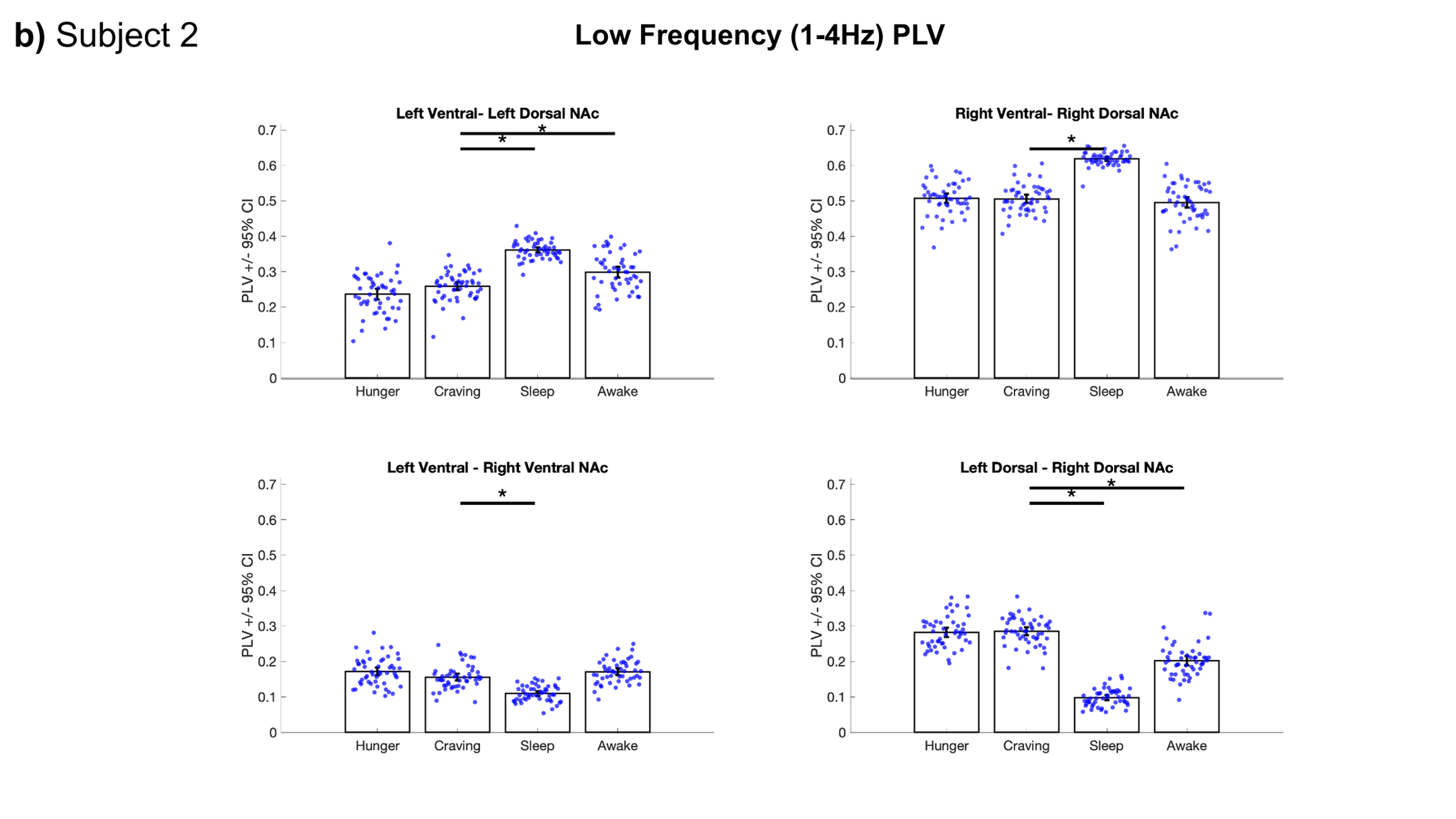

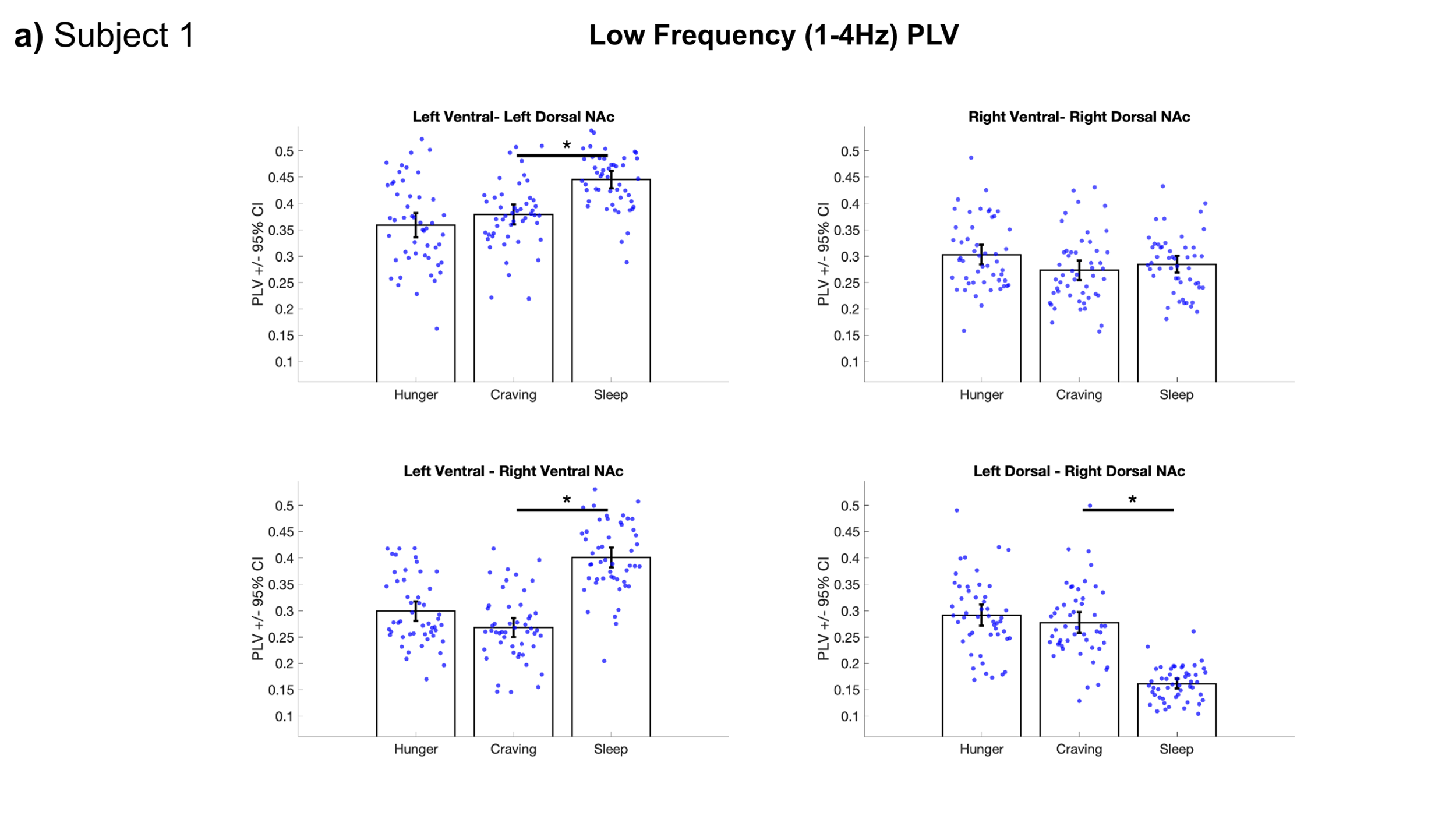


**Figure S2.** **Phase locking value (PLV) estimates between conditions using 60-second trial data.** The full 60 seconds of data preceding magnet swipes were analyzed for each trial. PLV estimates for low frequency (1-4 Hz; Subject 1: panel a, Subject 2: panel b) and high frequency (60-90 Hz; Subject 1: panel c, Subject 2: panel d) bands across hunger, craving, and sleep conditions for left ventral-left dorsal, left ventral-right ventral, right ventral-right dorsal, and left dorsal-right dorsal NAc connectivity pairs. Red lines indicate significant differences below a p-value statistical threshold of 0.002 (Bonferroni corrected for 4 connectivity pairs x 6 frequency bands). Blue circles indicate trial-level data.


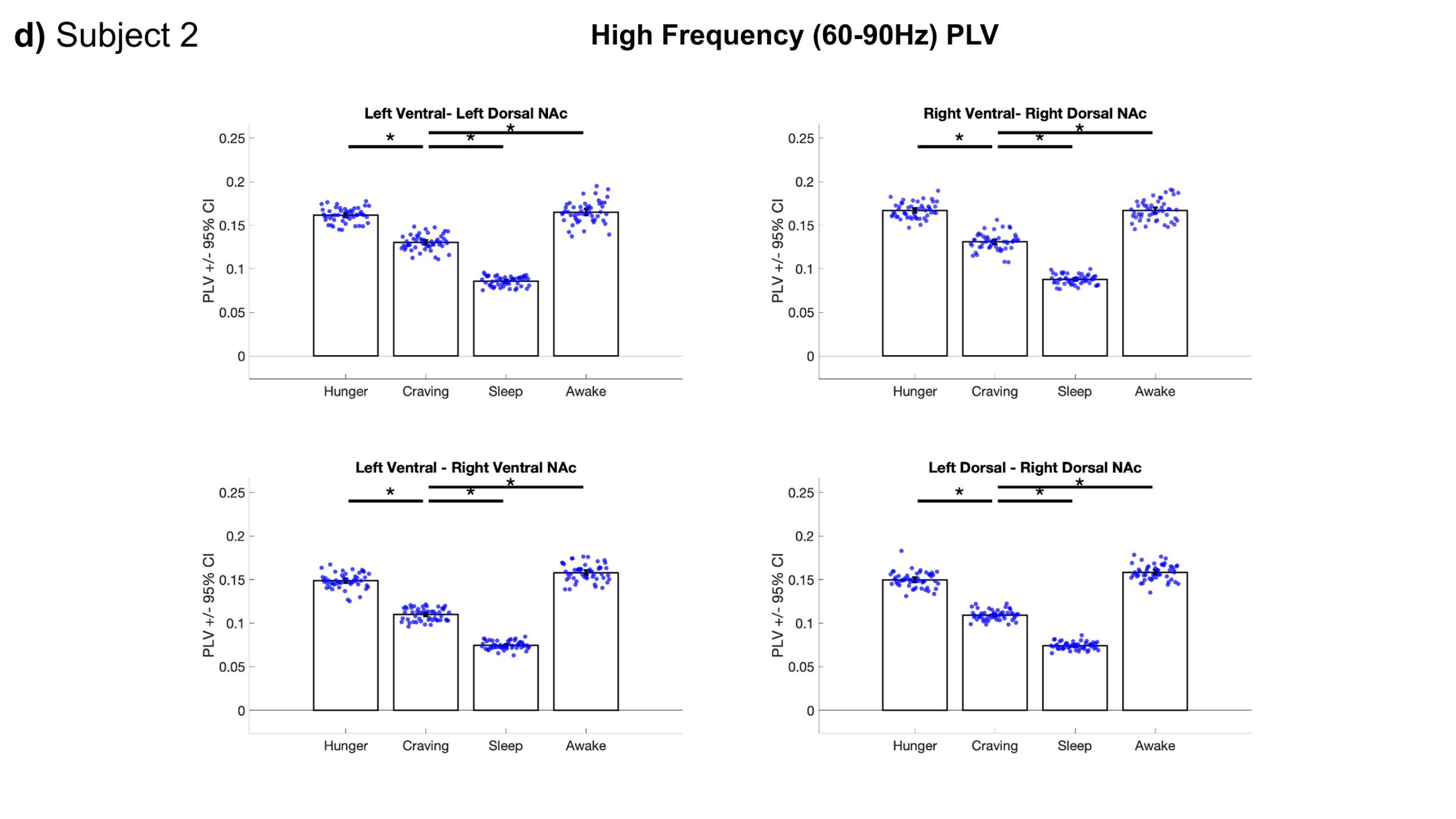

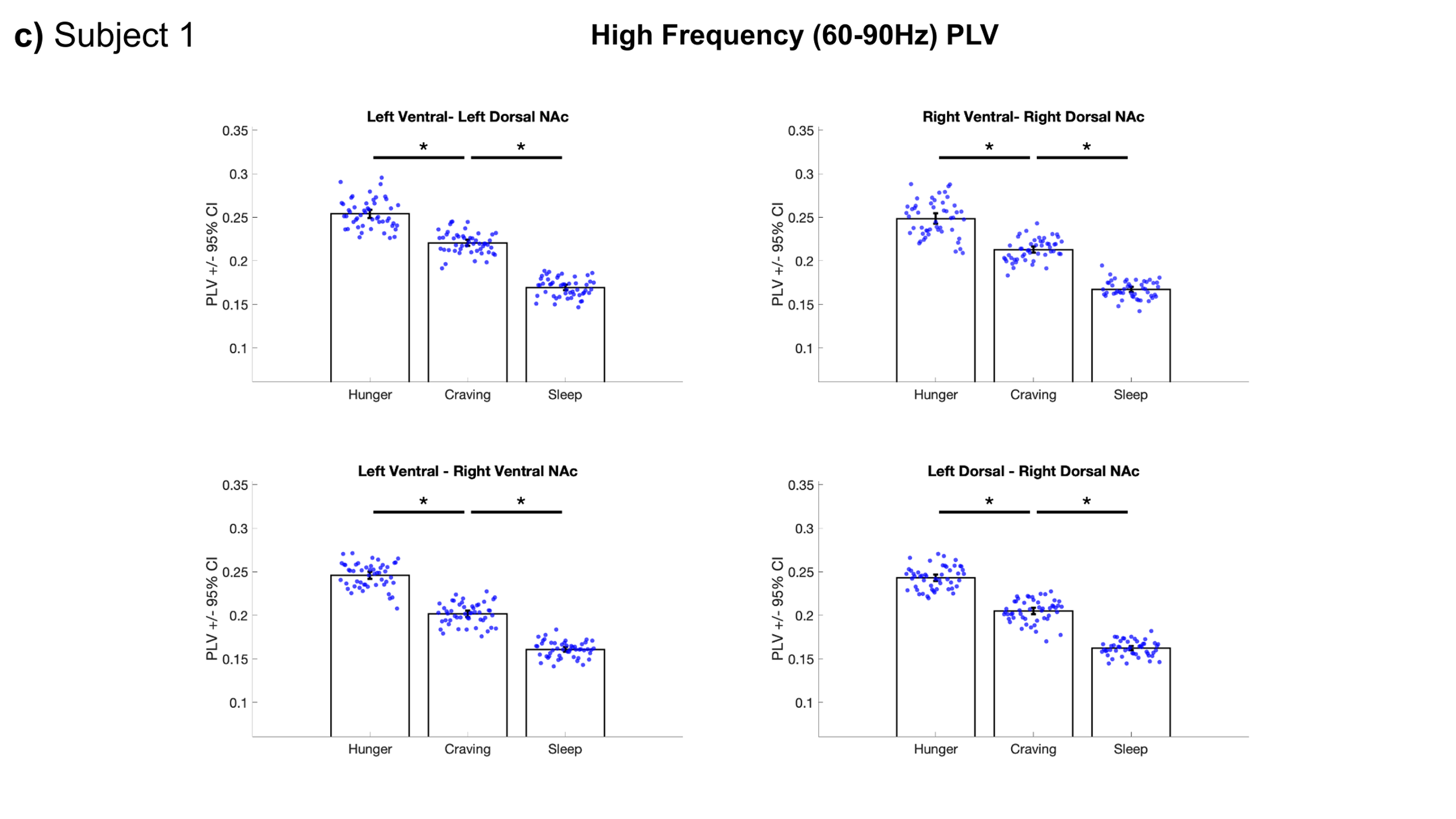

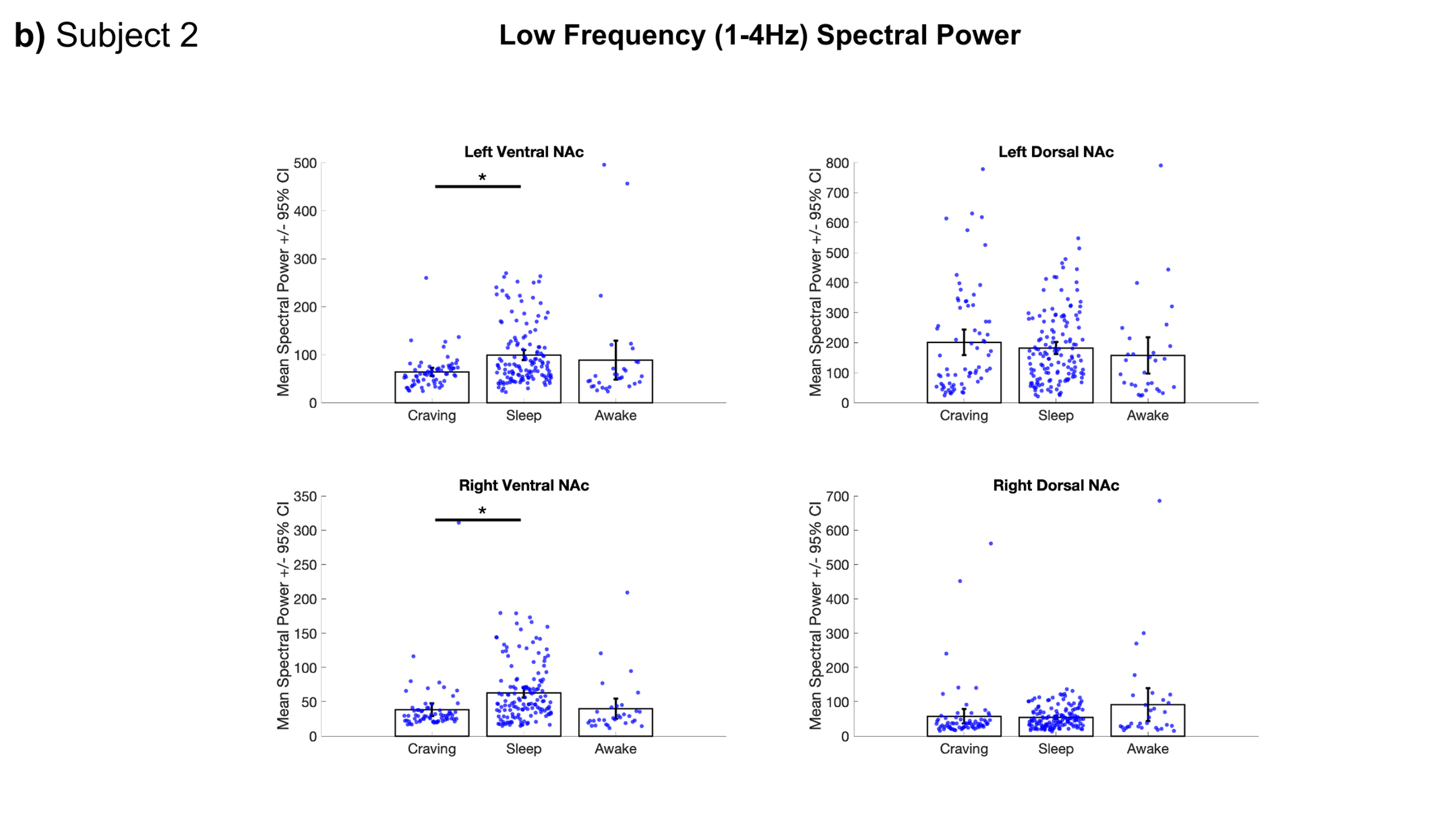

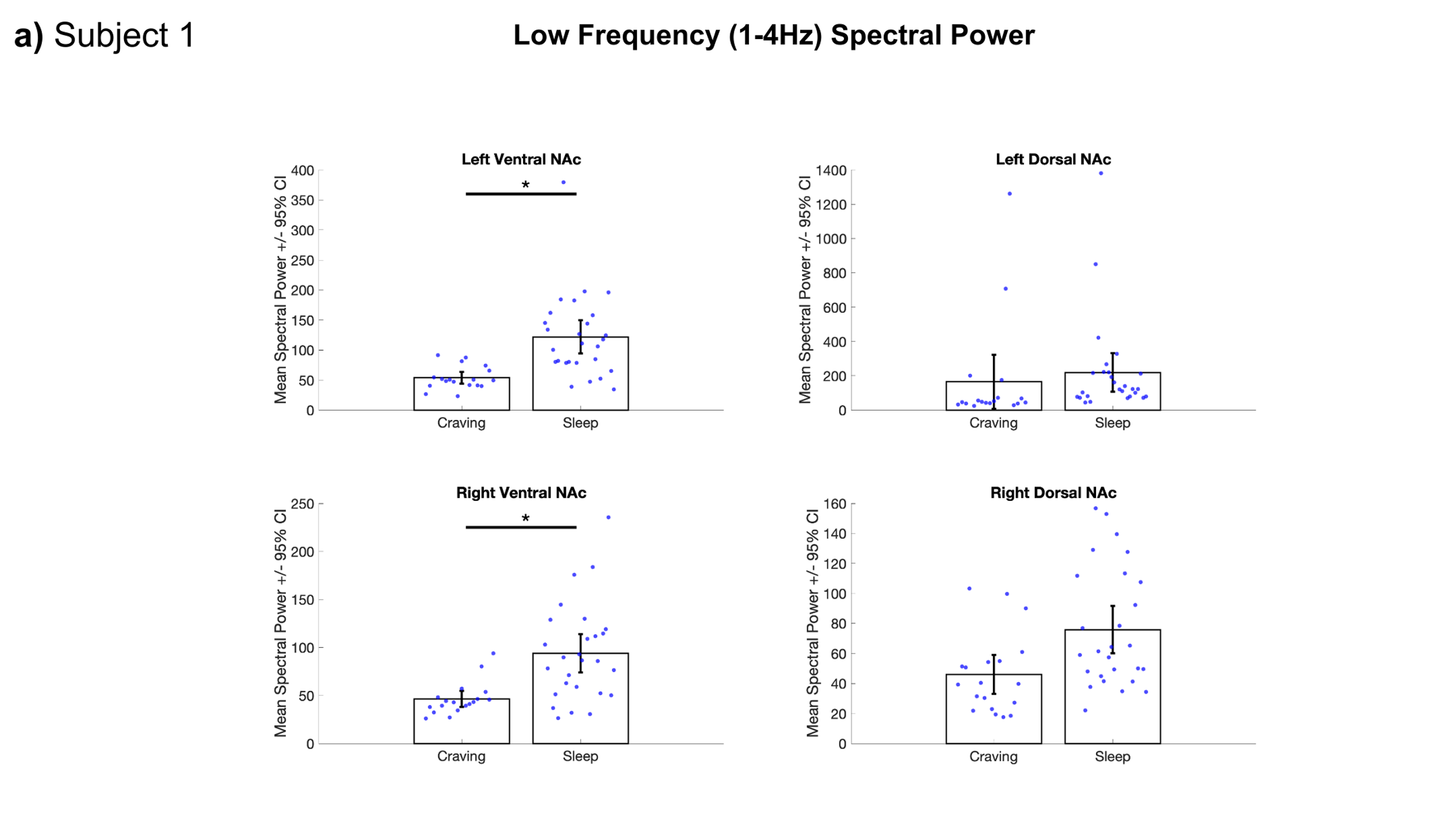


**Figure S3. Channel-level spectral power estimates between conditions.** Power band estimates for low frequency (1-4 Hz; Subject 1: panel a, Subject 2: panel b) and high frequency (60-90 Hz; Subject 1: panel c, Subject 2: panel d) across hunger, craving, and sleep conditions for left ventral, left dorsal, right ventral, and right dorsal NAc channels. Red lines indicate significant differences below a statistical threshold of .002 (Bonferroni corrected for 4 channels * 6 frequency bands). Blue circles indicate trial-level data. Power estimates were compared between conditions (craving vs. hunger, craving vs. sleep) using independent sample t-tests.


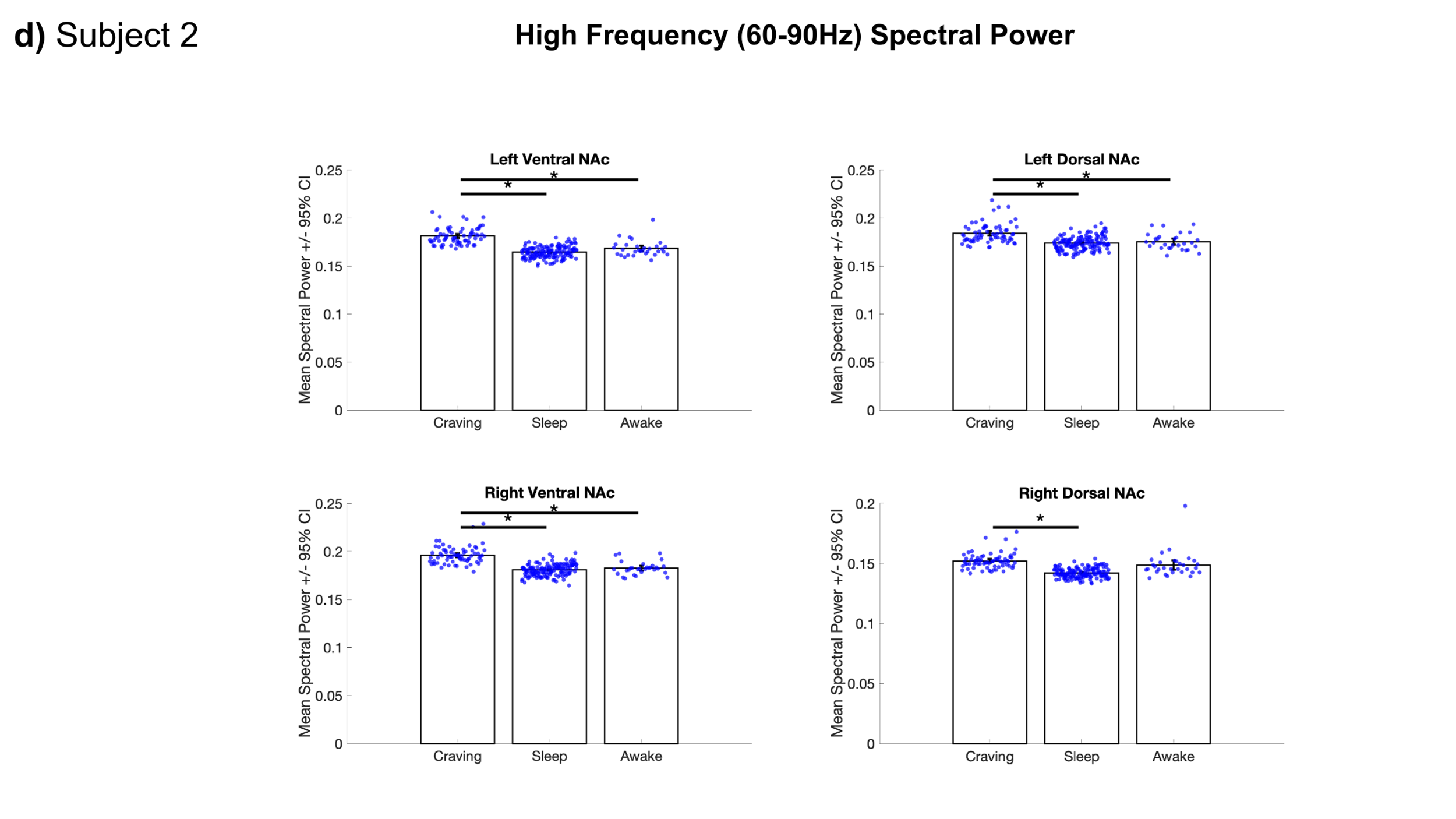

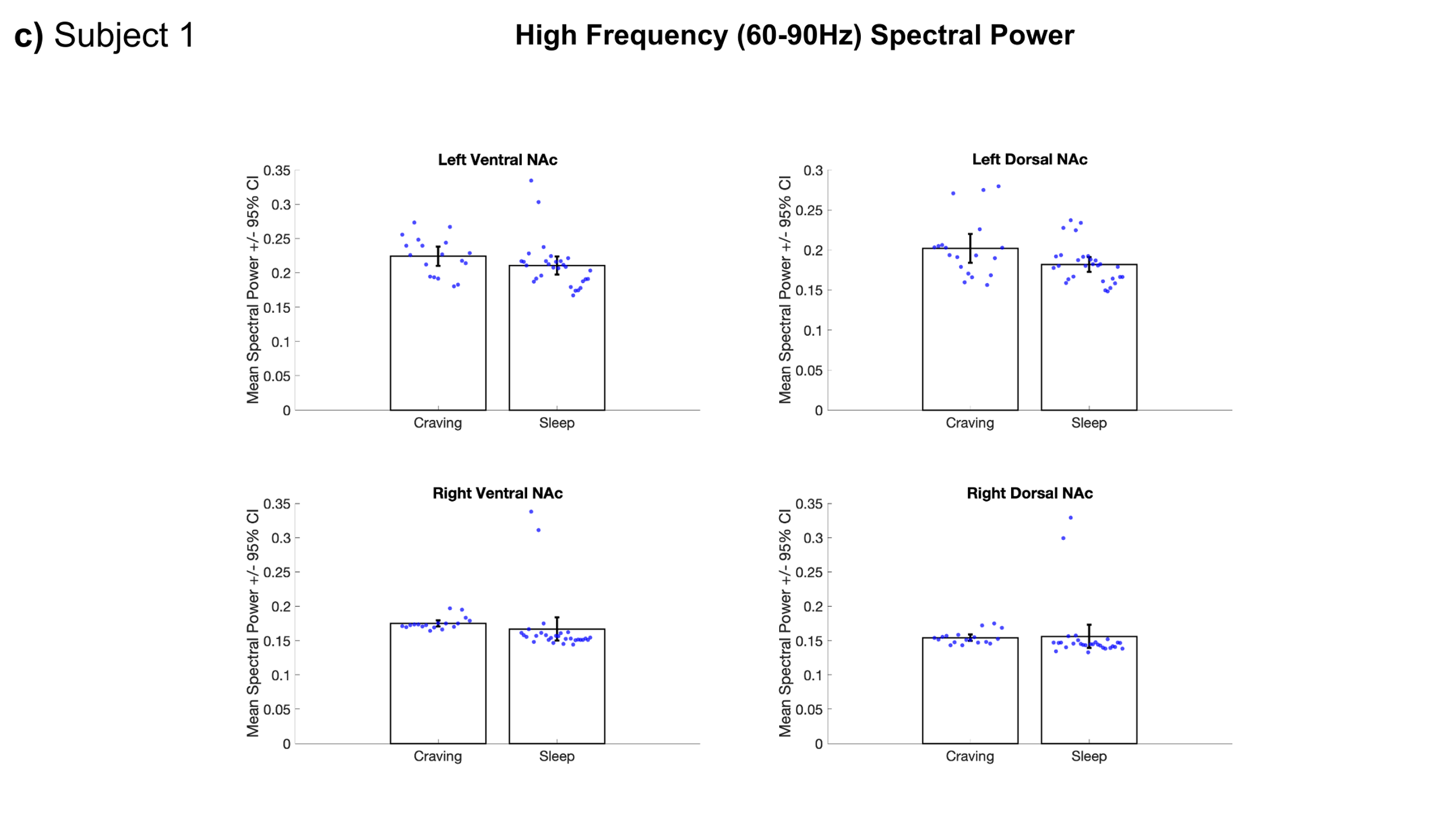

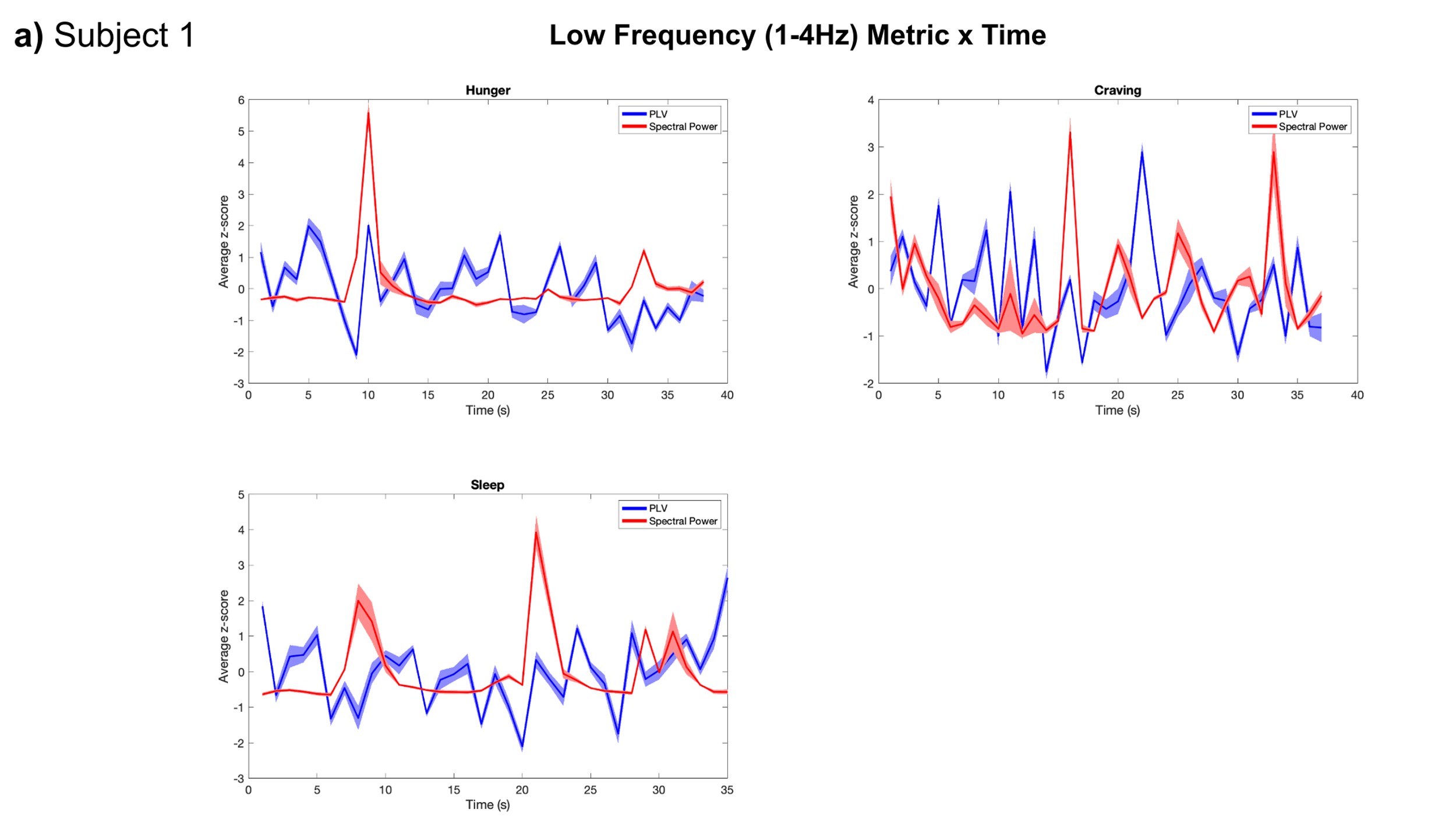

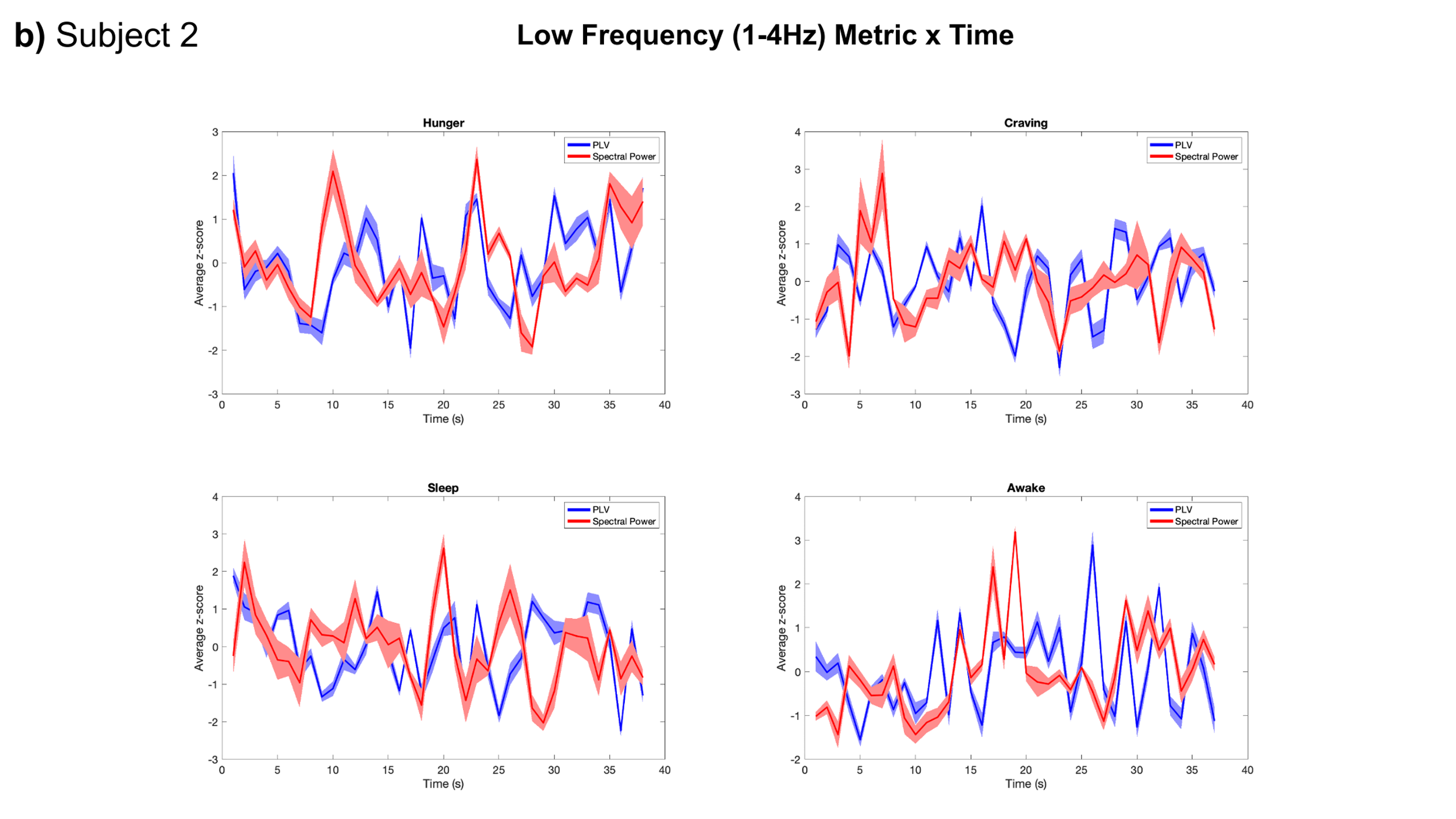

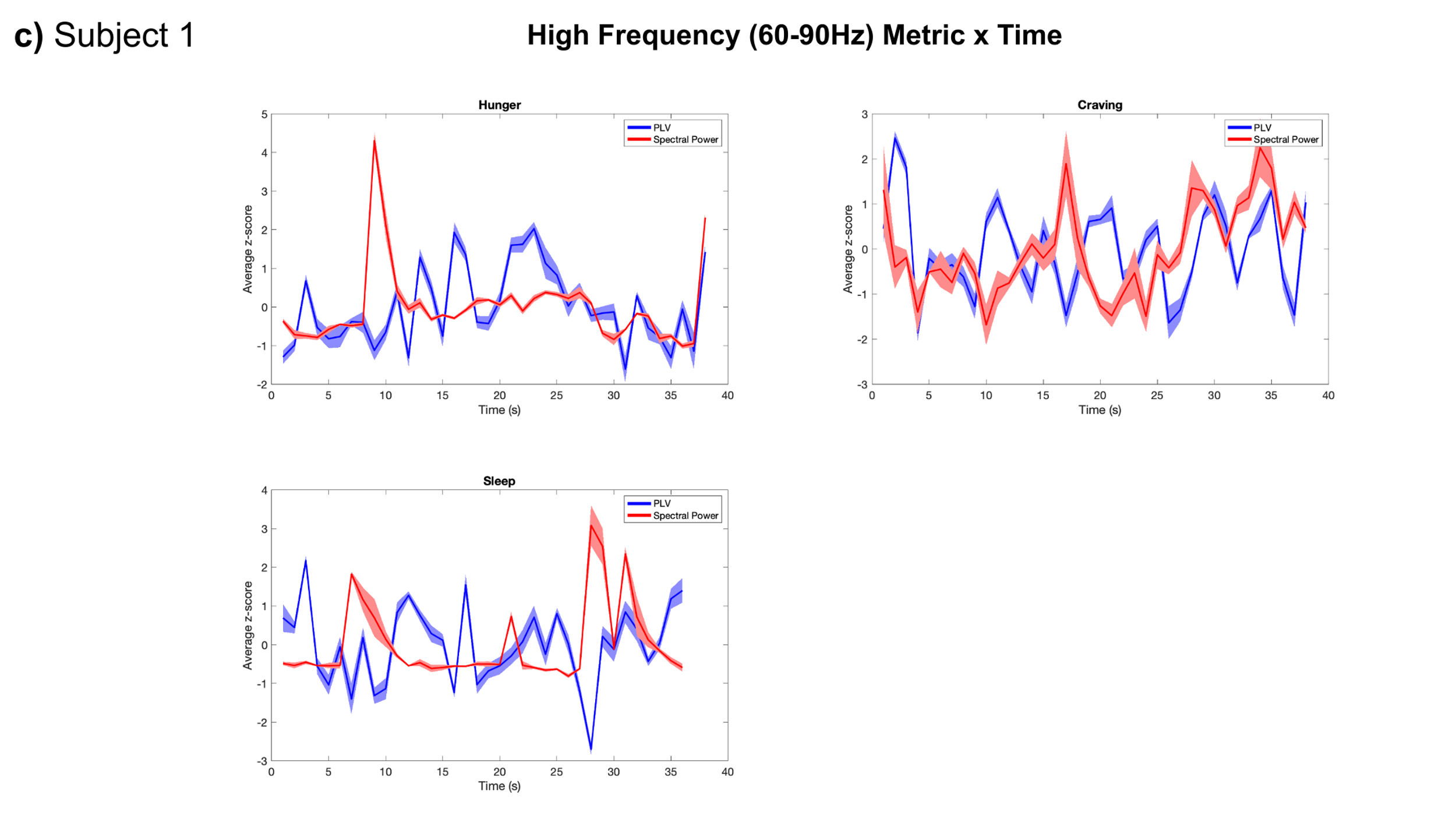

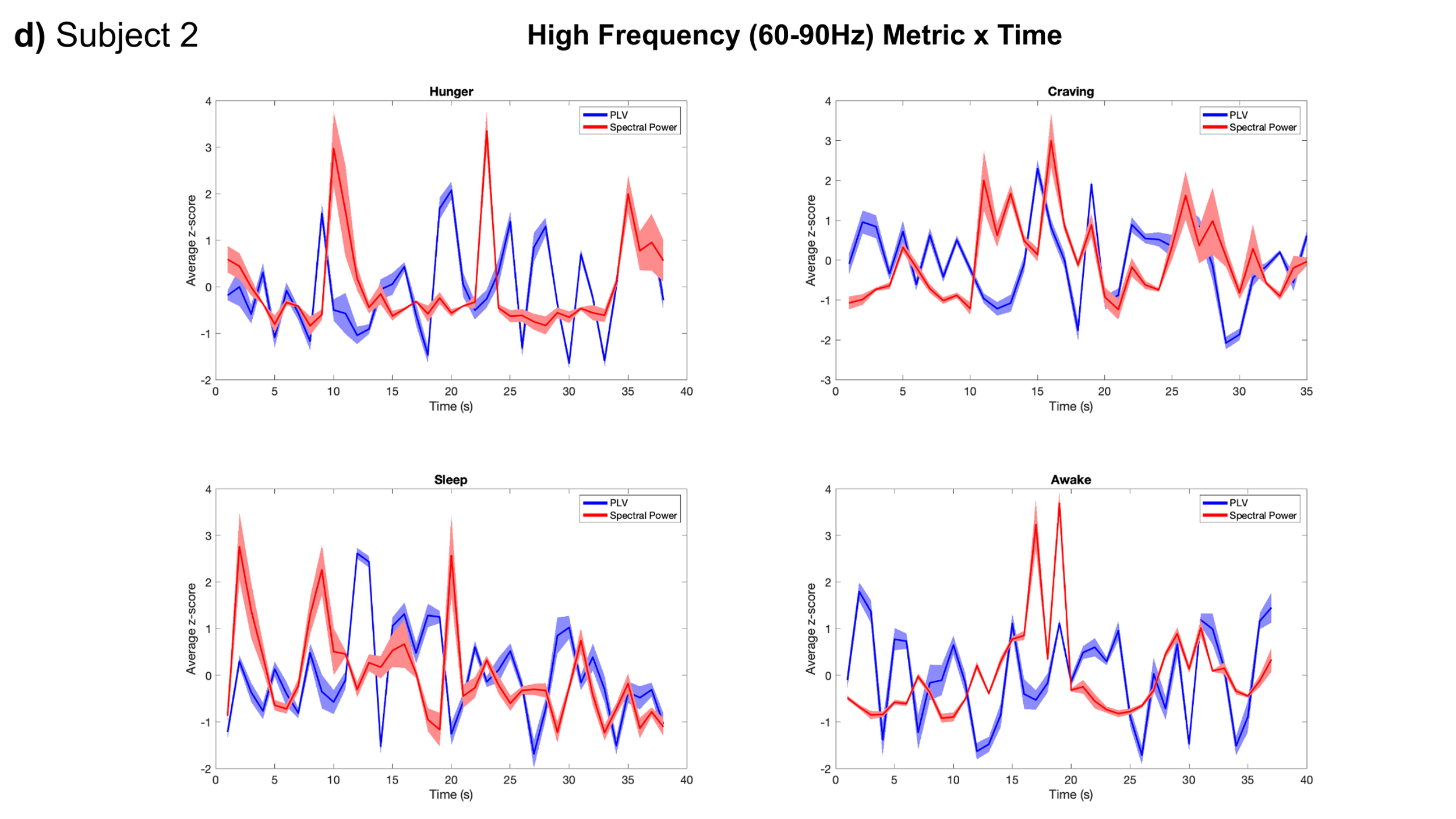


**Figure S4. Relationship between PLV and spectral power by condition and subject.** PLV across all trials and all connectivity pairs was averaged each second, z-scored, and plotted with mean and standard deviation. For comparison, spectral power across all trials and all channels was averaged each second, z-scored, and plotted with mean and standard deviation. Separate plots were generated for each condition (hunger, craving, sleep, and awake). Separate plots were generated by subject (subject 1: a and c, subject 2: b and d) and by low frequency (a and b) versus high frequency (c and d).

**Supplemental Tables**

Table S1 Stereotactic coordinates for contact localization


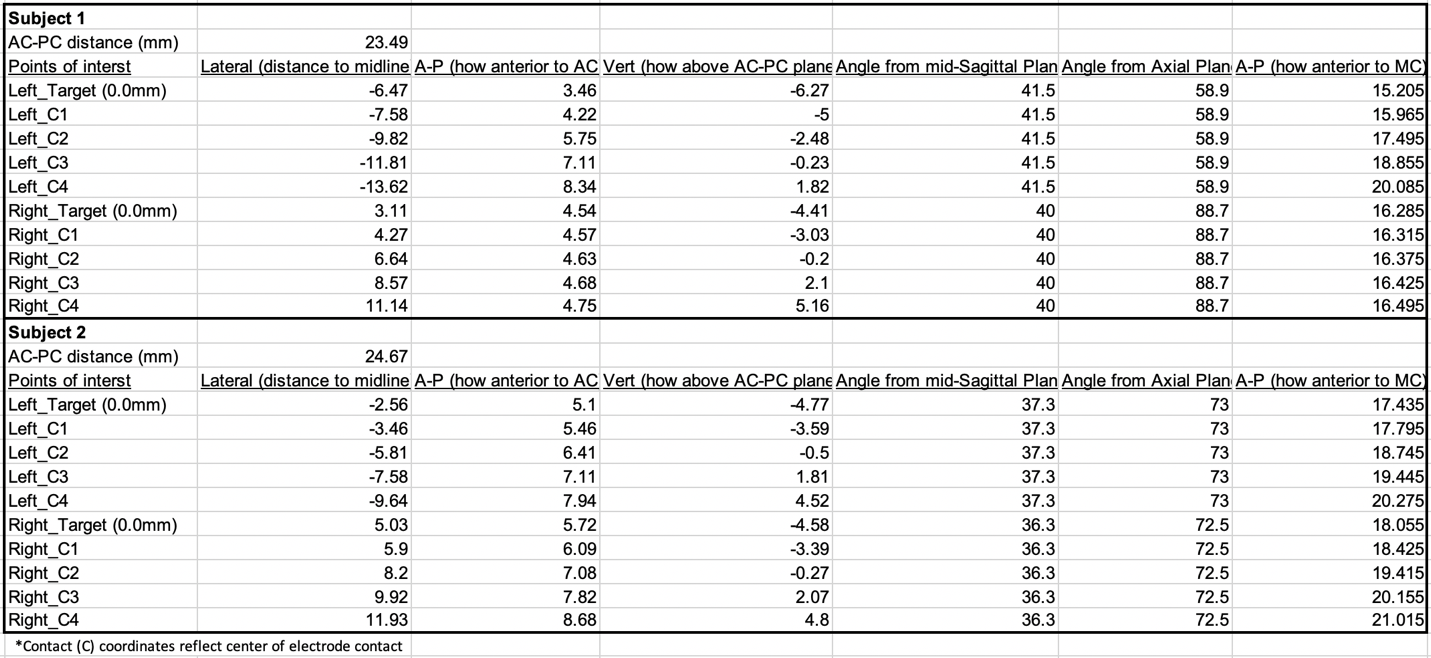


Table S2 P-values from PLV Condition Comparisons (t-test)


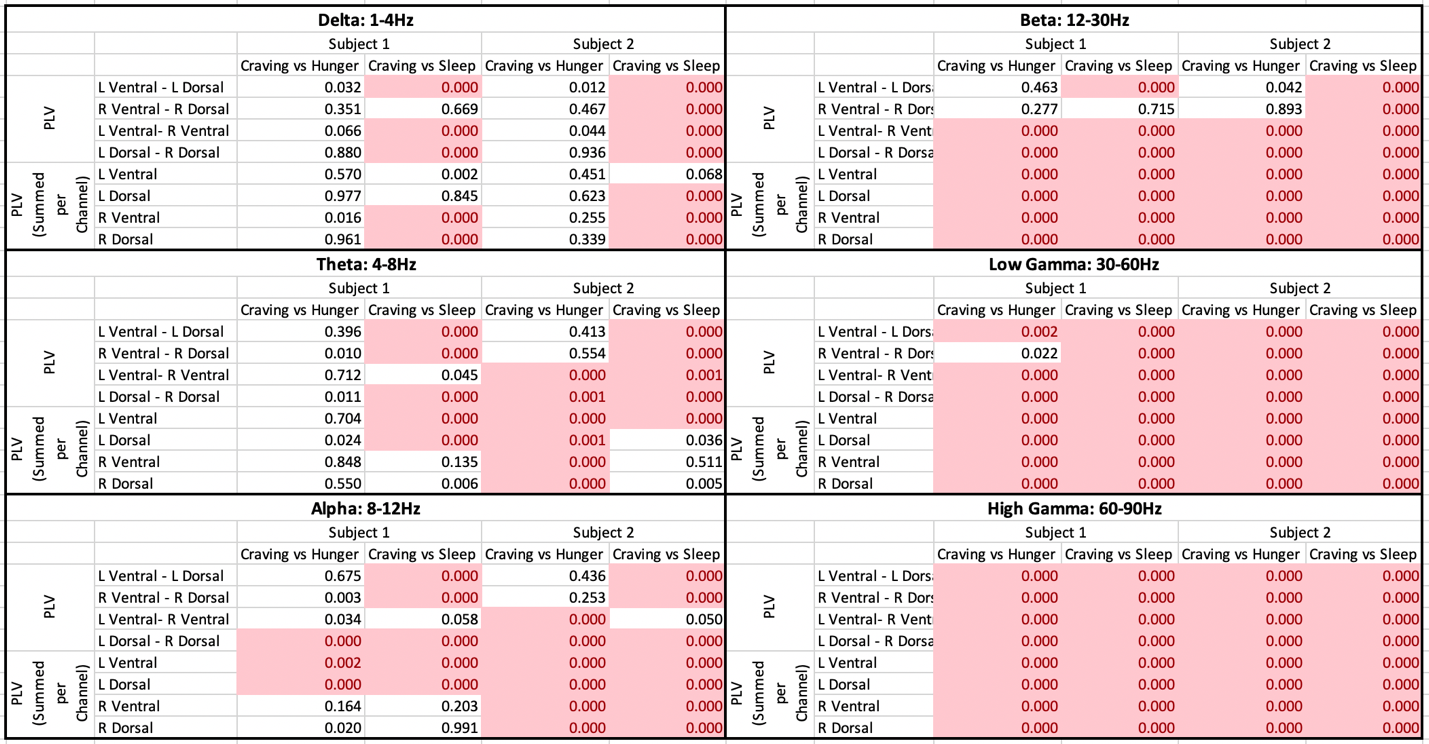


Table S3 T-values from PLV Condition Comparisons (t-test)


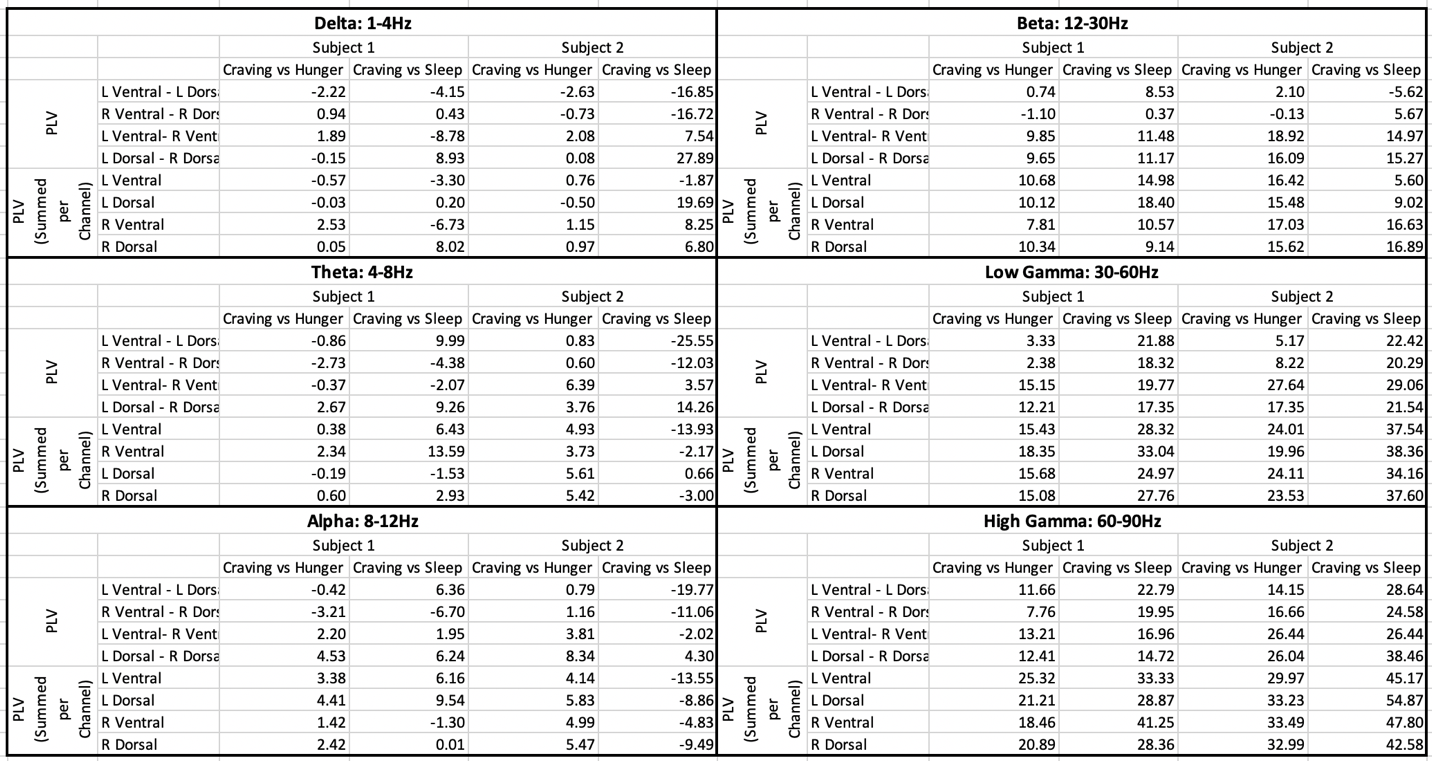


Table S4 P-values from Spectral Power Condition Comparisons (t-test)


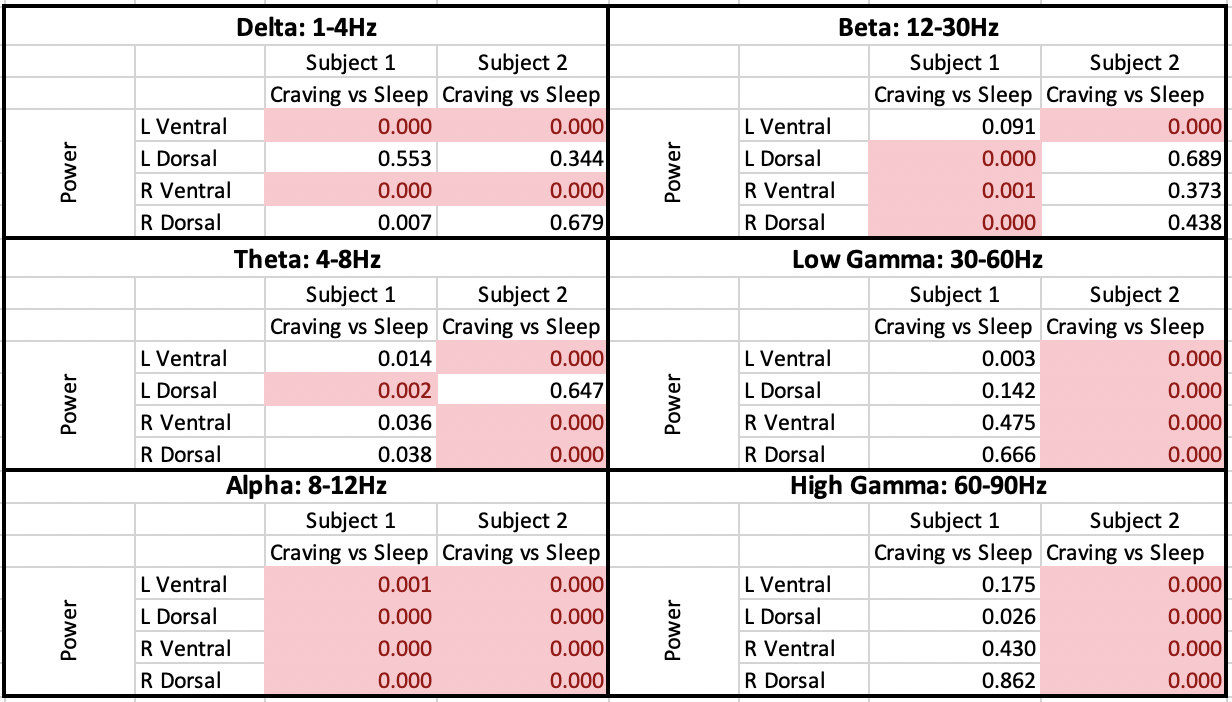


Table S5 T-values from Spectral Power Condition Comparisons (t-test)


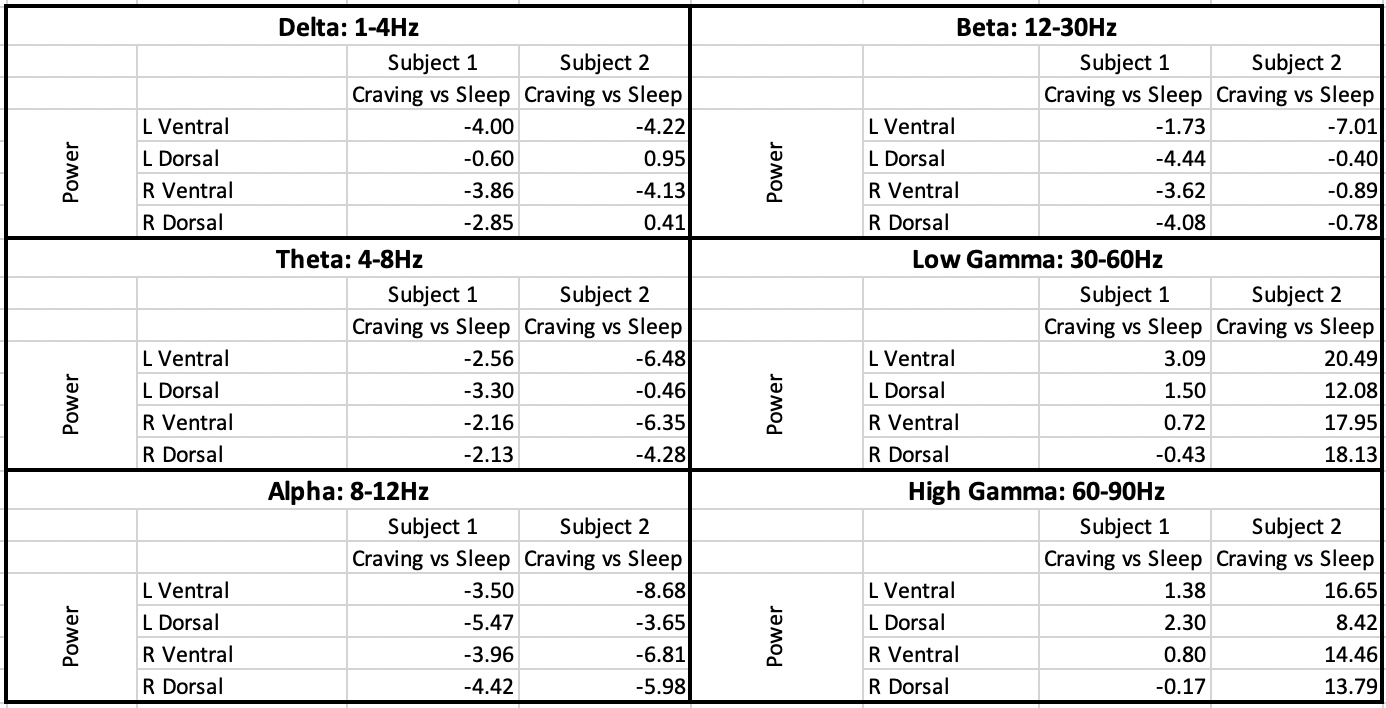


Table S6 Accuracies of Classifiers Trained on PLV Data (Using All Connectivity Pairs from a Frequency Band)

|  | **Subject 1** | **Subject 2** |
| --- | --- | --- |
| **Delta Band** |  |  |
| Craving vs. sleep | 74.68 ± 9.26 | 92.81 ± 4.31 |
| Craving vs. hunger | 51.88 ± 3.70 | 53.75 ± 3.27 |
| Craving vs. awake |  | 66.88 ± 6.71 |
| **High Gamma Band** |  |  |
| Craving vs. sleep | 98.44 ± 0.94 | 100 |
| Craving vs. hunger | 89.06 ± 2.57 | 97.50 ± 1.02 |
| Craving vs. awake |  | 97.19 ± 1.64 |

Table S7 Accuracies of Classifiers Trained on PLV Data (Using All Frequency Bands for a Given Connectivity Pair)

|  | **Subject 1** | **Subject 2** |
| --- | --- | --- |
| **L Ventral – L Dorsal** |  |  |
| Craving vs. sleep | 79.17 ± 10.52 | 98.75 ± 1.25 |
| Craving vs. hunger | 67.92 ± 9.85 | 67.92 ± 13.78 |
| Craving vs. awake |  | 82.08 ± 8.73 |
| **L Ventral – R Ventral** |  |  |
| Craving vs. sleep | 89.17 ± 4.23 | 81.25 ± 10.48 |
| Craving vs. hunger | 69.17 ± 12.30 | 77.08 ± 11.23 |
| Craving vs. awake |  | 82.08 ± 10.11 |
| **R Ventral – R Dorsal** |  |  |
| Craving vs. sleep | 70.42 ± 15.14 | 98.33 ± 1.10 |
| Craving vs. hunger | 61.67 ± 10.81 | 65.42 ± 16.24 |
| Craving vs. awake |  | 70.00 ± 12.83 |
| **R Dorsal – L Dorsal** |  |  |
| Craving vs. sleep | 93.75 ± 1.44 | 98.75 ± 0.72 |
| Craving vs. hunger | 67.50 ± 13.77 | 70.00 ± 15.07 |
| Craving vs. awake |  | 82.50 ± 10.90 |

Table S8 Accuracies of Classifier’s Trained on One Subject’s PLV Data, Tested on Another Subject

| **High Gamma Band** | **Train: Subject 1**  **Test: Subject 2**  **Test**  **Test:** | **Train: Subject 2**  **Test: Subject 1** |
| --- | --- | --- |
| **L Ventral – L Dorsal** |  |  |
| Craving vs. sleep | 50.00 | 50.00 |
| Craving vs. hunger | 50.00 | 50.00 |
| **L Ventral – R Ventral** |  |  |
| Craving vs. sleep | 47.50 | 50.00 |
| Craving vs. hunger | 50.00 | 50.00 |
| **R Ventral – R Dorsal** |  |  |
| Craving vs. sleep | 50.00 | 50.00 |
| Craving vs. hunger | 50.00 | 50.00 |
| **R Dorsal – L Dorsal** |  |  |
| Craving vs. sleep | 0.00 | 50.00 |
| Craving vs. hunger | 50.00 | 50.00 |
